# Supplementary material for: The Biosynthetic Pathway to the Pyrroloiminoquinone Marine Natural Product Ammosamide C
Source: J Am Chem Soc. 2025 Dec 5;147(50):46315–23. doi: 10.1021/jacs.5c15504 (PMC12715798; doi:10.1021/jacs.5c15504)
Supplement: Supplementary file 1 [file ja5c15504_si_001.pdf]

SUPPORTING INFORMATION

The Biosynthetic Pathway to the Pyrroloiminoquinone Marine Natural Product Ammosamide C

Josseline Ramos-Figueroa[1], Lingyang Zhu,[2] Matthew Halliman,[1] and Wilfred A. van der Donk[1*]

[1] Department of Chemistry and Howard Hughes Medical Institute, University of Illinois at Urbana-Champaign, Urbana, Illinois 61801, USA.

[2] School of Chemical Sciences NMR Laboratory, University of Illinois at Urbana-Champaign, Urbana, 61801, IL, USA.

*vddonk@illinois.edu

All MS and NMR data are deposited at:

Ramos Figueroa, Josseline; Zhu, Lingyang; van der Donk, Wilfred (2025), "Data associated with "The Biosynthetic Pathway to the Pyrroloiminoquinone Marine Natural Product Ammosamide C"", Mendeley Data, V1, doi: 10.17632/7hj55cr9zx.1

Materials and Methods

Plasmid construction and maintenance and protein overexpression were conducted using *Escherichia coli* DH10β and BL21 (DE3) strains, respectively. DNA sequences for primers and gBlocks were ordered from Integrated DNA Technologies (see Table S1). Q5 HF DNA Polymerase and deoxynucleoside triphosphate (dNTP) was purchased from NEB. NEBuilder HiFi DNA Assembly master mix was used for plasmid cloning following Gibson assembly protocols. Super DHB used as matrix in matrix-assisted laser desorption/ionization time-of-flight (MALDI-TOF) mass spectrometry (MS) was purchased from Sigma-Aldrich. C_{18} resin ziptip pipette tips were purchased from Millipore Sigma. The NuPAGE^{TM} 4%-12%, Bis-Tris, precast polyacrylamide gels, NuPAGE^{TM} LDS sample buffer (4X), NuPAGE^{TM} MES SDS running buffer (20X), and NuPAGE^{TM} sample reducing agent (X10) used for SDS-PAGE were purchased from Thermo Fisher. Precision Plus Protein^{TM} All Blue Protein ladder was purchased from Bio-Rad. Molecular grade water, and RNase/DNase free low-binding tubes were purchased from Fisher Scientific. DNA miniprep kits were purchased from Qiagen. Polymerase chain reaction (PCR) was performed using a Bio-Rad C1000 thermocycler. MALDI-TOF MS analysis was carried out in the mass spectrometry facility at the University of Illinois at Urbana-Champaign using a Bruker Daltonics UltrafleXtreme MALDI-TOF mass spectrometer. MALDI-TOF MS data were calibrated using Protein Calibration Standard I purchased from Bruker and processed using the software FlexAnalysis. Super-DHB matrix with a stock concentration of 25 mg/mL was mixed with samples in a 1 to 1 ratio and air-dried before MALDI-TOF-MS analysis. Structural predictions were performed using the AlphaFold3 server. Tandem mass spectrometry MS1 and MS2 data were obtained using an Agilent qTOF instrument equipped with an UPLC system. Two-dimensional (2D) nuclear magnetic resonance (NMR) experiments were recorded using a Bruker 500 MHz Avance III HD or 600 MHz Avance NEO spectrometer, both equipped with a 5 mm cryoprobe at 25 °C. The samples were in 90% H_2O and 10%D_2O. Ni-NTA resin for peptide and protein purifications was obtained from Thermo Fisher.

Peptide and protein expression and purification

Posttranslationally modified peptides were expressed by growing cultures of *E. coli* BL21 (DE3) cells co-transformed with plasmids bearing genes encoding N-terminal His-tagged AmmA*W (see

Figure S1) and modifying enzyme. pACYCDuet-1 was used to clone the gene sequences for His-tagged AmmA* in the first multiple cloning site (MCS); untagged PEARL sequences corresponding to $AmmB_3$ and $AmmB_1$ were cloned in the second MCS. $AmmB_3$ and $AmmB_1$ gene sequences were separated by a ribosomal binding site sequence (aaggagatataca). pCDFDuet-1 was used from a previously reported study[1] encoding an $AmmC_1$ homolog ($BhaC_1$) and Amm14 homolog (BhaG). For in vitro enzyme assays, genes encoding $AmmB_4$, Amm12, Amm13, Amm19, Amm20, Amm23, and *E. coli* ArgRS were cloned as His-tagged fusion constructs using a pET28b(+) vector. Plasmids were individually used to transform *E. coli* BL21 (DE3) electrocompetent cells. pET28b(+) harboring Amm3 was co-expressed with chaperones GroEL/ES expressed from a pACYC-Duet1 plasmid.[2,3] Single colonies were used to inoculate LB cultures with the corresponding antibiotics and grown overnight at 37 °C and shaken at 200 RPM. Overnight cultures were used to seed larger cultures that were grown until the OD600 reached 0.6-0.8 at which time expression was induced with IPTG to 0.4 mM final concentration. The cultures were then grown overnight at 18 °C and shaken at 200 RPM. Peptide expression to obtain modified peptide required up to 80 L of LB to obtain sufficient amounts for structural characterization. Cells were harvested in a centrifugal device at 5,200 ×*g* and the pellets were stored at −80 °C for future use or immediately resuspended in denaturing (6 M guanidinium hydrochloride, 50 mM HEPES, 300 mM NaCl, and 50 mM imidazole at pH 7.5; for peptides that were expressed insolubly) or native buffer (50 mM HEPES, 300 mM NaCl, and 50 mM imidazole, 5% glycerol at pH 7.5; for enzymes) containing Pierce protein inhibitor cocktail and 1 mM TCEP.

Peptide purification: Posttranslationally modified peptides were purified from cell paste resuspensions using denaturing buffer. All peptides were processed by sonication over ice with cycles of 2 s on and 5 s off for 5 min total and an amplitude of 50%. After cell lysis, centrifugation was performed at 50,000 ×*g* for 30 min and the supernatant was loaded onto a Ni-NTA resin previously equilibrated with start buffer (6 M guanidinium hydrochloride, 50 mM HEPES, 300 mM NaCl, and 50 mM imidazole at pH 7.5). After washing with 20 column volumes (CV) of wash buffer containing 50 mM HEPES, 300 mM NaCl, 50 mM imidazole, at pH 7.5 to remove non-specifically bound protein, the His-tagged peptide was eluted with 5 CV of 50 mM HEPES, 300 mM NaCl, 200 mM imidazole, at pH 7.5. The eluting solution was then used for HPLC purification or desalted using C18 ziptip and eluted in a 1.5 µL solution of Super-DHB of 25 mg/mL

concentration (dissolved in 80% CH_3CN (ACN)/20% H_2O/0.1% CF_3COOH (TFA)) for MALDI-TOF MS analysis.

Protein purification: All other proteins mentioned above were purified individually after resuspension of the cell pellet in 50 mM HEPES, 300 mM NaCl, 50 mM imidazole, 5% glycerol at pH 7.5. As for the peptide purification, lysis was performed by sonication over ice with 2 s on and 5 s off for total of 5 min on and amplitude of 40% magnitude. After centrifugation at 50,000 ×*g* for 30 min, the supernatant was loaded onto a Ni-NTA resin previously equilibrated with 50 mM HEPES, 300 mM NaCl, 50 mM imidazole, 5% glycerol at pH 7.5. After washing with 20 CV of 50 mM HEPES, 300 mM NaCl, 50 mM imidazole, 5% glycerol at pH 7.5 to remove non-specifically bound proteins, the His-tagged protein was eluted with 5 CV of 50 mM HEPES, 300 mM NaCl, 200 mM imidazole, 5% glycerol at pH 7.5. The excess imidazole content was then removed by concentration and buffer exchange into 50 mM HEPES, 300 mM NaCl, 5% glycerol at pH 7.5 using the appropriate MWCO Amicon centrifugal device. Amm3 purification followed the same protocol, but different start, wash, and elution buffers were used. Start and wash buffer contained 20 mM NaH_2PO_4, 500 mM NaCl, and 50 mM imidazole at pH 7.5 with 5% glycerol, and elution buffer consisted of 20 mM NaH_2PO_4, 500 mM NaCl, and 200 mM imidazole at pH 7.5 with 5% glycerol, while storage buffer (after imidazole removal) contained 5.0 mM K_3PO_4 and 5% glycerol at pH 8.0.

High-performance liquid chromatography (HPLC) purification of 6 to 9

Peptides obtained from Ni purification were acidified with a 10% trifluoroacetic acid (TFA) solution to a final concentration of 0.1 to 2% TFA to precipitate any coeluting protein from the overexpression protocol. The sample was then passed through a 0.45 µm syringe filter and injected directly into an Agilent 1260 Infinity II HPLC system for purification. The peptide was purified using a preparative scale column, VP NUCLEODUR C18 HTec, 5 µm, 250 x 10 mm with the following gradient: 0-5 min isocratic 2% acetonitrile (ACN), 5-40 min 2%-60% ACN, 40-42 min 60%-100% ACN; mobile phase contained 0.1% TFA as reported in previous work.[1] Fractions were collected manually and were submitted for MALDI-TOF MS analysis by combining 1 to 1 with a 25 mg/mL solution of SDHB (prepared as mentioned above). Fractions containing the desired peptide (usually bright purple in color) were combined and lyophilized. The dried purple solids

were then used as substrates for *in vitro* studies, further purified on an analytical scale column for structure elucidation, or digested for LC-MS or NMR analysis when required.

When an additional round of purification was required, peptides were redissolved in deionized water, passed through a 0.45 µm filter, and injected directly onto an analytical scale Agilent 1260 Infinity HPLC system. A Vydac 218TP C18 column, 5 µm, 250 x 4.6 mm was used for peptide purification using the following gradient: 0-10 min isocratic 2% ACN, 10-30 min 2%-60% ACN, 30-32 min 60%-100% ACN; mobile phase contained 0.1% TFA. Desired peptide was identified by MALDI-TOF MS analysis performed on each collected fraction.

Dipeptide **8** and compound **9** required a different column for purification. After the *in vitro* reaction of peptide **7** with Amm12/Amm13, and with Amm12/Amm13 and Amm19, respectively, dipeptide **8** and compound **9** were purified by a first round of purification using a Vydac 218TP C18 column using the following gradient: 0-10 min isocratic 2% ACN, 10-30 min 2%-60% ACN, 30-32 min 60%-100% ACN; mobile phase contained 0.1% TFA. After detecting an impurity in the purified peptide by NMR spectroscopy, a second round of purification was performed by using a Chromosorb Varian C18 column using the following gradient: 0-10 min isocratic 2% ACN, 10-30 min 2%-60% ACN, 30-32 min 60%-100% ACN; mobile phase buffered with 0.1% TFA.

When the samples were used for NMR experiments or LC-MS/MS analysis, HPLC-purified **7** to **9** were first subjected to trypsin or chymotrypsin proteolytic digest in a 1 to 20 w/w ratio. The samples were then acidified to 0.1 to 1% TFA, filtered through 0.45 µm membrane or centrifuged at 13,000 RPM for 5 min, and injected onto the HPLC for further purification. The fragment containing the C-terminal modifications were then eluted with the same column and gradient as indicated above for the analytical scale purification. Fractions were collected manually and submitted for high resolution (HR) MS analysis for peptide fragment identification and NMR analysis.

LC-MS analysis of compounds 7 to 10

After trypsin digestion (for **7**) or after *in vitro* reactions performed to obtain **8** to **10**, each solution was desalted using a Pierce™ C18 spin column. Briefly, the spin column was activated with 200 µL of 60% ACN with 0.1% formic acid (FA) and rinsed three times with 200 µL of 0.1% FA. The sample was loaded onto the equilibrated column and the column was washed three times with 200 µL of 0.1% FA. Then, the compounds of interest were eluted using 25 µL of 60% ACN with 0.1%

FA, twice. The elutant was lyophilized, redissolved in 25-50 μL of water, and passed through a 0.45 μm membrane to remove any insoluble material. The sample was then injected onto an Agilent 1260 Infinity II with a 6545 LC/Q-TOF mass spectrometer detector. For tryptic fragment **7**: A Kinetex® 2.6 μm C8 100 Å column, 150 x 2.1 mm was used following the gradient: 0-3 min isocratic 5% ACN, 3-13 min 5%-95% ACN, 13-15 min 95%-5% ACN; mobile phase containing 0.1% FA. For compounds **8** to **10**: A Poroshell EC-C18 column, was used following the gradient: 0-3 min isocratic 5% ACN, 3-13 min 5%-95% ACN, 13-15 min 95%-5% ACN; mobile phase containing 0.1% FA. A DualAJS ESI detector was utilized with the following parameters, Gas temperature of 325 °C, drying gas of 13 L/min, nebulizer of 35 psi, sheath gas temperature of 275 °C, sheath gas flow of 12 L/min, nozzle voltage of 500 V and MS-TOF fragmentor set at 175 V.

AmmB$_4$ *in vitro* experiments

In a 200 μL tube, a solution containing a final concentration of 50 mM HEPES, 25 mM KCl, and 15 mM $MgCl_2$ pH 7.6, 2 mM DTT, 5 mM L-arginine, or $^{13}C_6$-labeled L-arginine, 6 mM ATP, 10 U thermostable inorganic pyrophosphatase (TIPP), 10 U superase RNAse inhibitor, 3 ng/μL of tRNA (*E. coli* $tRNA^{Arg}$), 20 μM of the corresponding aminoacyl-tRNA synthetase ArgRS, 5 μM of PEARL AmmB$_4$, and 15 μM of peptide substrate (intermediate **6**) was incubated for 4 h at 37 °C in a heating block. After this time, the modified His-tagged peptide was isolated using Ni agarose resin. The eluted His-tagged peptide was buffer exchanged into 50 mM HEPES pH 7.5 and further cleaved with trypsin or chymotrypsin for ESI-MS/MS analysis.

Amm12/Amm13 and Amm19 *in vitro* experiments

HPLC-purified **7** was incubated with Amm12/Amm13 and Amm19 separately to a final protein concentration of 25 μM at 37 °C for 4 h. After this time, the solutions were desalted using C18 ziptip and eluted in a 1.5 μL solution of Super-DHB of 25 mg/mL concentration for MALDI-TOF MS analysis. Additionally, **7** was also incubated with Amm12/Amm13 and Amm19 together and after incubation for 4 h, the two reaction mixtures were desalted for MALDI-TOF analysis and HR MS/MS using Pierce™ C18 spin column.

The same procedure was repeated for intermediates in the pathway preceding **7** and the reaction mixtures were analyzed on a MALDI-TOF spectrometer.

Amm3 *in vitro* experiments

HPLC-purified **9** was incubated in a mixture containing 5 µM FAD-containing Amm3, 10 µM MibS, 50 mM NaCl and 2 mM NADH in a 50 mM HEPES buffer at pH 8.0 overnight in a shaker at 37 °C and shaking at 200 RPM. After this time, the solutions were desalted using a Pierce™ C18 spin column for HR MS/MS analysis. Similarly, the reaction was repeated in a mixture containing NaBr and analyzed by HR MS/MS.

To test the substrate scope of Amm3, several tryptophan (Trp) analogs (Figures S16 and S17) were evaluated as substrates for Amm3. L-Trp was shown to be a substrate and thus the chlorination site was studied. Scaled-up 50-µL reactions containing 1 mM Trp were set up ten times and an FAD-recycling system was used in an attempt to push the reaction towards higher yields of the chlorinated Trp after two-day incubation. The reaction mixture contained 25 mM HEPES, 50 mM NaCl, 1 mM NAD+, 5 mM phosphite, 1 mM Trp or Trp analog, 20 µM Amm3, 25 µM MibS, 30 µM phosphite dehydrogenase (PTDH),[4] and 0.2 µg/µL catalase. After the incubation time, the samples were processed in two ways. For Trp analogs that were not retained by Pierce™ C18 spin columns, protein was precipitated by adding an equal volume of ice cold ACN and centrifuged at 13,000 RPM for 5 min. The supernatant was then analyzed by HR MS/MS. In all other cases, desalting was performed using a Pierce™ C18 spin column and the flowthrough analyzed by HR MS/MS.

For the reaction mixtures containing Trp as a substrate, after scale-up, the reaction was acidified with TFA to a final concentration of 2%. After centrifugation at 13,000 RPM for 5 min, the supernatant was purified using an analytical scale HPLC as described above.

Amm20 *in vitro* experiments

HPLC-purified **9** and **10** were incubated in a mixture containing 20 µM Amm20, 50 mM L-glutamine, 2 mM ATP, and 1 mM $MgCl_2$ in 50 mM HEPES buffer containing 150 mM NaCl at pH 8.0 overnight at 37 °C. After this time, the solutions were desalted using a Pierce™ C18 spin column for HR MS/MS analysis. Similarly, the reaction was repeated in a mixture containing lysates of untagged Amm20 and analyzed by HR MS/MS.

Amm23 *in vitro* experiments

HPLC-purified **9** and **10** were incubated with Amm23 separately to a final protein concentration of 25 µM and 20 mM SAM in 50 mM HEPES buffer with 150 mM NaCl at pH 8.0 at 37 °C for 4 h. After this time, the solutions were desalted using a Pierce™ C18 spin column for HR MS/MS analysis.

Table S1. List of primers and double stranded DNA fragments applied in this study.

**DNA Name**	**Sequence**
JRF_AW_B3_B1.VF	AAGGAGATATACAatggctgccacctcag
JRF_AW_B3_B1.VR	tttctttaccagactcgattagacagtttcccaacgtaa
JRF_AW_B3_B1.GF	gaaactgtctaatcgagtctggtaaagaaaccgc
JRF_AW_B3_B1-2.GR	ctgaggtggcagccatTGTATATCTCCTTttagggcgccgccac
JRF_gb_Amm16	ATGACTGCACCTGTCCTTCATGCCGTTGTTACGGGAGCGCCGCCAGCCGGT<br>GATGTTGGACGCCTCGTAGCTTTGGCTGGGGCGGATGGATGGGGAACAC<br>ACCTGGTGGCATCGCCTAGTGCTCGGCCGTTCCTTGATCTTCCGAGATTAG<br>CGGCAGCAACGGGGCATCCTGTTCGGACTGATTACCGGCAACCGGGCGAT<br>CCTTGGCCTCTCCCACCACCTACCGCCGTGGTGCTGGCCCCAGCTACAGGT<br>AATACCTTAGCCAAATGGGCTGCGGGCATATCAGACACTTTAGCGCTCGG<br>TCTGCTCGTGGAAGCTGTTGGCCAGGGTACGCCAGTTGTGGCGGCACCAT<br>TCTCAAATACGGCACATCTGGCACATCCAGCGGTAGCGGAAGCAATCACT<br>CGGCTGCGCAGCTGGGGTGTTACTGTTCTCACCGGACCAGAGGTTTGTCC<br>ACCACACCCGCCGGGACCTGCAGCCGCGCATGCAGAACGTTTTGGTTGGG<br>ACGCTGTTTGGGCAGCAGTTACTGGTCATCCTCACCTGGCCGGTGCACGG<br>GCTGCCGCTCCAGCTCCGGATCCAATCCCTCAAGAGCAGCCCGCTTAG
JRF_gene_fragment_Amm19	ATGTCACATAGAGATCGATGCCGGGCCGCTGTTGAACGCCGTCGTAGCAC<br>GTTATTGGAACTGTCCCACTCGCTTCACGCAGAGCCTGAGACAGCCTTTGA<br>TGAGCATCGGAGTGCGGCCAAGATTGCCGATTTACTTGAAGGTGAGGGAT<br>TTGCGGTGGAGCGTGGTACTGGCGGGTTAGCAACCGCCTTGACTGCGACA<br>TGGGGAGACGGCGAGCTCGTTGCCGGAGTTTTGGCTGAGTATGACGCCTT<br>GCCGGGGATCGGGCACGCATGCGGCCATAACGTCATAGCTGCAGCTAGT<br>GTTGGTGCGGCACTGGCCTTACGAGAGGTTGCAGCCGACCTCAACCTGAC<br>AGTCCGATTAATTGGTACTCCGGCTGAGGAATCCGGTGGCGGGAAGGTTC<br>TGCTTCTTGAGCGGGGCGCGTTTGATGATTTAGCTTTTGCGTTATTGATCC<br>ATCCGTCCCCTGACGAGATCTGCGCACCAAGAACATTGGCTGTTACCGATT<br>TGGAAGTCACTTATCGGGGCCGCGCAGCACACGCGAGCTTCGCTCCCCAC<br>CAAGGGGTAAATGCGGCAGACGCACTTACTGTAGCGCAAGTAGCTATTGG<br>ATTAGCGCGTCAACATCTCGAGCCCCACCAGATGGTGCACGGGATTACTA<br>CGCACGGCGGAGACGCGCCCAACATAGTACCGGATCGAACCGGCGCGGT<br>TTATTACTTACGTGCCCGATCAGCTGAATCCCTGAGCCGGTTAGAGCATCG<br>CGTCCGGGATTGCTTACGTGCCGGTGCTGTCGCTACCGGCTGCACAGAAG<br>AGATCCGTAGAGCAGCACCGGCGTACACAGAACTGGTGTCCGATGTTCGT<br>TTGACCGCAGCATATCGAGAAGCTATTACGGCCCTCGGGCGTGTACCGCT

	TGATGAGGAAACCGAAGCAAGACTTGTCACACCTGCTAGTACTGATATGG<br>GCAATGTTTCTCAGGTTCTTCCAGCGATTCAACCGTCGATAGCGGTCGATG<br>CCCGCGGTGCGGTTAACCATCAACCAGGCTTTGCGGAAGTTTGTGCAGGT<br>CCCTCAGGCGATGCTGCTGTCCTGGATGGTGCGGTGGCGCTTGCCTGGAC<br>CGCTGTTGCTGCTGCCAGTGATCCTGCACATCGTGTGGCATTATTAGACGG<br>CGTCCGCGCGAGACGTTGTGGCGCCGTTCGTTGA
JRF_gene_fragment_Ecoli_ArgRS	ATGAACATACAAGCCTTATTGTCCGAGAAAGTTCGGCAAGCTATGATAGC<br>CGCCGGTGCACCTGCGGACTGCGAACCTCAAGTGCGACAGTCCGCTAAGG<br>TGCAATTCGGGGACTACCAAGCCAACGGCATGATGGCTGTTGCTAAGAAA<br>CTGGGTATGGCTCCGCGTCAACTTGCCGAGCAGGTCTTGACACACTTGGA<br>TCTCAACGGCATCGCCTCGAAAGTGGAGATCGCTGGACCAGGCTTCATTA<br>ATATTTTCCTGGACCCAGCCTTTCTGGCCGAACATGTTCAACAAGCTTTGG<br>CTTCTGACCGGTTGGGAGTTGCAACACCGGAGAAGCAAACCATAGTAGTC<br>GATTACTCTGCACCGAACGTAGCAAAGGAGATGCATGTTGGTCACTTGCG<br>CTCGACGATCATCGGGGACGCCGCGGTTCGAACACTGGAGTTTCTTGGCC<br>ACAAAGTGATACGTGCGAACCACGTGGGCGACTGGGGTACTCAATTTGGC<br>ATGCTTATTGCGTGGTTAGAAAAGCAACAGCAGGAGAATGCTGGCGAAAT<br>GGAATTAGCCGACTTAGAGGGGTTCTACAGAGATGCGAAGAAACATTACG<br>ACGAGGACGAGGAGTTCGCCGAGCGTGCTCGCAATTACGTTGTTAAGTTG<br>CAGAGTGGTGACGAGTACTTCCGTGAGATGTGGCGGAAGCTTGTTGATAT<br>CACAATGACTCAAAACCAAATAACTTATGATCGTCTGAACGTCACGCTGAC<br>GCGGGATGACGTGATGGGTGAGAGCTTATACAACCCAATGTTGCCAGGC<br>ATAGTTGCAGACCTTAAAGCAAAGGGGCTGGCTGTGGAGAGCGAAGGTG<br>CCACTGTGGTATTCCTTGACGAGTTTAAGAATAAGGAAGGTGAGCCGATG<br>GGCGTTATCATTCAGAAGAAAGATGGCGGCTATCTTTATACAACAACCGA<br>CATTGCTTGTGCAAAGTACCGTTACGAGACCCTTCACGCCGATCGGGTTTT<br>ATATTATATCGATAGCCGTCAGCACCAACATTTGATGCAAGCATGGGCTAT<br>TGTGAGAAAAGCTGGATACGTGCCGGAATCCGTCCCGCTTGAACACCACA<br>TGTTCGGGATGATGCTCGGAAAGGACGGGAAGCCGTTTAAGACCCGAGC<br>CGGCGGAACGGTTAAACTTGCCGACCTCTTGGACGAAGCTTTAGAACGTG<br>CGCGCCGTCTGGTGGCTGAAAAGAACCCGGACATGCCGGCTGATGAACT<br>GGAGAAACTTGCGAATGCTGTTGGCATTGGGGCTGTTAAGTACGCAGACT<br>TGTCTAAGAATCGTACCACCGATTACATCTTCGATTGGGATAATATGCTTG<br>CGTTCGAGGGGAATACAGCACCTTACATGCAGTACGCTTACACTCGAGTTC<br>TTAGCGTGTTTCGCAAGGCGGAGATCGATGAGGAGCAATTGGCTGCGGC<br>GCCCGTGATTATTCGGGAAGATAGAGAGGCCCAATTGGCAGCTCGGCTGC<br>TCCAGTTCGAGGAGACGTTGACGGTAGTAGCCCGTGAGGGCACTCCACAC<br>GTTATGTGTGCGTATTTGTACGACCTCGCAGGCTTGTTCTCAGGTTTCTAC<br>GAACACTGCCCGATTCTGAGTGCTGAAAATGAGGAAGTGCGTAACTCGCG<br>ACTTAAGCTGGCCCAGTTGACTGCAAAGACGCTGAAGCTGGGACTGGATA<br>CGTTGGGGATCGAGACTGTGGAGAGAATGTAA
JRF_gene_fragment_Amm22	ATGGCCGAGAAATCAAAACCGGGTAGCAAGCCTTACCGGAGAGACCTGC<br>GAGAACGGTTAAAGAGCCTCGGATTTGCTGACCATCAGATCGACGACGTT<br>GTCACAGCGGAGTTGGTACAAATGTGTCACATCCGCCCGCGTACCGCCAG<br>AAGACTTGCAAGAGAGTTAACTTTGGACGAGGTTGCGACGCGTTACTCCA<br>GTCTGCGTAATGATGCTGAGTCACGCATGCGCGGCTCGCGGATCTGGGAC<br>TACGAGCAATGGCCGAACCGTGGCGTGCGGCCTACAGTCCACACACTGCG<br>TACATTGGCCGAGGTTTACGGTACAACTTGGGTAGAGCTCGTAGACATTG

	AGGATCTCCAACACATGCCGGAAGAAGACCGGGAACTTTACCACCTTCGG GTATCAGGCGGGCCCCCTGACACACGTGCTGCCCCTACCGCTCCCCTTCCC CCGAGTCTGCCGGCTGCACCGCCGGCCTGTGCACCTCCTCCAGCCCATGCG CACGCTCCGGCGCCAGACCGTCCTCGTCCCCGCCCAGCTCCAGCTCAACCT GACCCATCTGGTCCGGCCGGAACTGCCGAACTCATACGTACTGGTGCTGA ACACGCGTTAGGAGTGGCTCGCAGCCTTAGTACCACGAATGTAGATTCCTT GACTTTACACCAATTAGAGCAAGAGTTAGCCAGTACCTCTCGGTCGTATGT TCACAGTTCCCCATATCCGTTGTATTTTCGGTTGGCACAGCTGGGCAACCA CATTGCTCAATTACTGCCAGGTCGGCAGCGCCCGTCTCAAACCCGCAGACT GAATGCACTGGCCGCCCGGTGCTCTGCCTTGTTAGCGTGGGTATGCGATG ATTTAGGCGCCGCAGCAGCCGCGCACGACCACGCGTGGGCAGCATGGAT CTATGCATCTCACAGCGAAAGCGAGGACGCACAGCGCTGTGTAAGTCTGG TCCGTTCTCGCTTGGCATTTTCATCTGGAGACTTTATGGAGAGTGCCCGAA TTGCAGATAGCGCCTGTAGTGGCGTCCCCCAAGATAGTGTCGGATCACAT TTGTTGCTGCGACAAGCGTTGGCCTGGGCCCGTGCAGGGCAACACCACAA GGTACACGAGGCCCTTCGGGATTGGAAGGCAAGAGGAGAGGAAGGTCC GCTCGGAGCTGGTGACGGCATATTTGAACTTCAACAAGCGCAGCAAAGAT ATTTCGTAGGGGCCGCGCTGCTTGATATTTTAGACGTTGAGCATGGTCTGG ACGAGCTGCGTACCGCCATCGATCTGTTCAACCGTACGCCCGAGGATCTG CGCTCATACGTGGAACACTCACTGAGCCATATTGGAACGGCGAAAGCCTT CGTGGTCCTCGGCGATGTACAAGAGGCATCCAAGGTAGTTGAGCCTGTAT TCGACCTCGCACCCGAACGTCGCGTACGCGCAATCCTGTCTAGTCTCCGCG AGCTGGTCGCGTTCACGGCCGGCAGACCTTCCTACGGAAGTCAACTGGCT CGGGAGTTGTCTGAGCGAATTGACGAGTTCGCATCCCATGCTATCACTCGC CGGTTACACTCAGTTGTGTAA
JRF_pRSF_Amm22_VF	ATCCCATGCTATCACTCGCCGGTTACACTCAGTTGTGTAATGCAGGTCGAC AAGCTTGCG
JRF_pRSF_Amm22_VR	GTCTCTCCGGTAAGGCTTGCTACCCGGTTTTGATTTCTCGGCCATCGGATC CTGGCTGTG
JRF_pRSF_Amm19_VF	GGATGGCGTACGAGCCCGCCGGTGTGGTGCAGTCCGGTAGTGCAGGTCG ACAAGCTTGCG
JRF_pRSF_Amm19_VR	TCTTCGGCGTTCTACGGCGGCTCGGCAACGGTCGCGCATCGGATCCTGGC TGTGGTGATG
JRF_pRSF_Amm16_VR	CGCTCCCGTAACAACGGCATGAAGGACAGGTGCAGTCATCGGATCCTGGC TGTGGTGATG
JRF_pRSF_Amm16_VF	TCCAGCTCCGGATCCAATCCCTCAAGAGCAGCCCGCTTAGTGCAGGTCGA CAAGCTTGCG
JRF_AWB3B1.F	CTGGGGTGTGTTGTTTGGCCAGCCGAAGAAGGACGCTAAtcgagtctggtaaa gaaaccg
JRF_AWB3B1.R	GCGCTGTGCGTGTGGAAGTCATTGTATATCTCCTTttagacagtttcccaacgtaa tgc
JRF_B4_AWB3B1.F	tttggcattacgttgggaaactgtctaaAAGGAGATATACAATGACTTCCACACGCA CAG
JRF_B4_AWB3B1.R	ggcgttcaaatttcgcagcagcggtttctttaccagactcgaTTAGCGTCCTTCTTCGGC
JRF_pET28_A20CtHis.F	ATATTTGGCTGCGTCATTACAGTCCGGAGATCGCCGTACACCACCACCACC ACCACCACC
JRF_pET28_A20CtHis.R	GGTATATCTCCTTCTTAAAGTTAAACAAAATTATTTCTAGAGG

JRF_A20_CtHis.F	CTCTAGAAATAATTTTGTTTAACTTTAAGAAGGAGATATACCATGTGTGGC ATAGTCGGC
JRF_A20_CtHis.R	CAGCCGGATCTCAGTGGTGGTGGTGGTGGTGGTGGTGGTGTACGGC GATCTCCGGAC
JRF_pAB1B3B4.R3	ctcactataggggaattgtgagcggataacaattccccatcttag
JRF_pAB1B3B4.F	Cctgtgtttcggacatcgg
JRF_gDNA-A20-2F	ggcagcgtggacggcgtcagagaagggagcggacatatgATGTGCGGAATAGTCGGTT GG
JRF_gDNA-A20-2R	cggggatctaagcttggctgcagtcaatgatgatgatgatgatgGACCGCGATCTCCGGG
JRF_pET28-ArgS_R	TGGATACGTTGGGGATCGAGACTGTGGAGAGAATGTAACATATGGCTAG CATGACTGGTG
JRF_pET28-ArgS_F	GCCGAACTTTCTCGGACAATAAGGCTTGTATGTTCATGTGATGATGATGAT GATGGCTGC
JRF_Ec_tRNA-Arg-ACG-r	mUmGGTGCATCCGGGAGGATTCGAACCTCCGACCGCTCGGTTCGTAGCC GAGTACTCTATCC
JRF_Ec_tRNA-Arg-ACG-f	AATTCCTGCAGTAATACGACTCACTATAGCATCCGTAGCTCAGCTGGATAG AGTACTCGG
pET28a(+)_His_Amm23	ATGCCGGCACCTACTGACCTGCGCCTCAGAGAACTGGTAACGGGATACCG CACGTCCCAAGTCGTGTATGCCACTGTAAAACTGGGCCTGTTTGATCTGCT GGGTGCGCGCCCTCGCACGGCGGCCGAACTCGCCGAAGCCGGAGGTGCA GATCCAGATGCACTGGCACGTTTTGTACGAGCCCTGACAGCGGAAAGAAT ACTGCAGTCCACAGCGGACGGTGTATTATCACTGACACCACTCGGAGAAA GACTTCGTAGTGATGCACCTGGTGGGCTGGCGGCGTGGGTGGTAACGAG CTGCGAAGAGCAGTTCTTTGCATGGGGCCATGCGGTGCATACGCTGCGAA CGGGCCAATCAGCATTTGAAAAAGCCCATGGAGTCGGGTTCTGGGAGCAT TTACGCTCTGATGCTAAAGCGGCAGAAAGCTACGATGCAGGCATGGCCGT GTCAGCGGGTGAGGCATGCGATCTTATTGTGTCTGCCGGGGGCATCGATC GAGCTCGACATGTTGTTGATGTGGGTGGTGGCCGGGGTGCGCTCGCACG GCGGCTTCTTGATACCTTCCCACATGCGCGGGTCACCGTTCTGGACCTTCC CGAGGTCGTAGAACGTACGCGCGCGAGTTTTGCACCTGATCCAGTTCCGG AGCGCTTAGGCCTGGTACCCGGATCTTTTTTAGACGGCGTCCCTGCCGGG GGGGATGTTTACGTACTGTCACGTGTACTGGCCGATTGGGATGACGCTCA TGCCGCTAGAATCCTGGCGTCTTGCCGGCGCGCCATGACACCCGGGGGGC GCCTGCTGATTGCTGAGGGACTGACTCGGGATGATGCACCCGCAGGGGC CCGCGGCTTGCTCGATTTGCATTTACTGTTGCTGTTCGGTGGACGTGAGCG CACAGTGACCGGCCTTACCGCTCTGCTGAGCGGTGCCGGATTTGCGTTGG TGTCCGTGACGGGCGCTGACAACGGAGGCATTTCGCTGCTGACGGCCGTA GCTCGGTAACTCGAGCACCACCACCACCACCACTGAGATCCGGCTGCTAAC AAAGCCCGAAAGGAAGCTGAGTTGGCTGCTGCCACCGCTGAGCAATAACT AGCATAACCCCTTGGGGCCTCTAAACGGGTCTTGAGGGGTTTTTTGCTGAA AGGAGGAACTATATCCGGATTGGCGAATGGGACGCGCCCTGTAGCGGCG CATTAAGCGCGGCGGGTGTGGTGGTTACGCGCAGCGTGACCGCTACACTT GCCAGCGCCCTAGCGCCCGCTCCTTTCGCTTTCTTCCCTTCCTTTCTCGCCAC GTTCGCCGGCTTTCCCCGTCAAGCTCTAAATCGGGGGCTCCCTTTAGGGTT CCGATTTAGTGCTTTACGGCACCTCGACCCCAAAAAACTTGATTAGGGTGA TGGTTCACGTAGTGGGCCATCGCCCTGATAGACGGTTTTTCGCCCTTTGAC GTTGGAGTCCACGTTCTTTAATAGTGGACTCTTGTTCCAAACTGGAACAAC

	ACTCAACCCTATCTCGGTCTATTCTTTTGATTTATAAGGGATTTTGCCGATT<br>TCGGCCTATTGGTTAAAAAATGAGCTGATTTAACAAAAATTTAACGCGAAT<br>TTTAACAAAATATTAACGCTTACAATTTAGGTGGCACTTTTCGGGGAAATG<br>TGCGCGGAACCCCTATTTGTTTATTTTTCTAAATACATTCAAATATGTATCC<br>GCTCATGAATTAATTCTTAGAAAAACTCATCGAGCATCAAATGAAACTGCA<br>ATTTATTCATATCAGGATTATCAATACCATATTTTTGAAAAAGCCGTTTCTG<br>TAATGAAGGAGAAAACTCACCGAGGCAGTTCCATAGGATGGCAAGATCCT<br>GGTATCGGTCTGCGATTCCGACTCGTCCAACATCAATACAACCTATTAATTT<br>CCCCTCGTCAAAAATAAGGTTATCAAGTGAGAAATCACCATGAGTGACGA<br>CTGAATCCGGTGAGAATGGCAAAAGTTTATGCATTTCTTTCCAGACTTGTT<br>CAACAGGCCAGCCATTACGCTCGTCATCAAAATCACTCGCATCAACCAAAC<br>CGTTATTCATTCGTGATTGCGCCTGAGCGAGACGAAATACGCGATCGCTGT<br>TAAAAGGACAATTACAAACAGGAATCGAATGCAACCGGCGCAGGAACAC<br>TGCCAGCGCATCAACAATATTTTCACCTGAATCAGGATATTCTTCTAATACC<br>TGGAATGCTGTTTTCCCGGGGATCGCAGTGGTGAGTAACCATGCATCATC<br>AGGAGTACGGATAAAATGCTTGATGGTCGGAAGAGGCATAAATTCCGTCA<br>GCCAGTTTAGTCTGACCATCTCATCTGTAACATCATTGGCAACGCTACCTTT<br>GCCATGTTTCAGAAACAACTCTGGCGCATCGGGCTTCCCATACAATCGATA<br>GATTGTCGCACCTGATTGCCCGACATTATCGCGAGCCCATTTATACCCATA<br>TAAATCAGCATCCATGTTGGAATTTAATCGCGGCCTAGAGCAAGACGTTTC<br>CCGTTGAATATGGCTCATAACACCCCTTGTATTACTGTTTATGTAAGCAGA<br>CAGTTTTATTGTTCATGACCAAAATCCCTTAACGTGAGTTTTCGTTCCACTG<br>AGCGTCAGACCCCGTAGAAAAGATCAAAGGATCTTCTTGAGATCCTTTTTT<br>TCTGCGCGTAATCTGCTGCTTGCAAACAAAAAAACCACCGCTACCAGCGGT<br>GGTTTGTTTGCCGGATCAAGAGCTACCAACTCTTTTTCCGAAGGTAACTGG<br>CTTCAGCAGAGCGCAGATACCAAATACTGTCCTTCTAGTGTAGCCGTAGTT<br>AGGCCACCACTTCAAGAACTCTGTAGCACCGCCTACATACCTCGCTCTGCT<br>AATCCTGTTACCAGTGGCTGCTGCCAGTGGCGATAAGTCGTGTCTTACCGG<br>GTTGGACTCAAGACGATAGTTACCGGATAAGGCGCAGCGGTCGGGCTGA<br>ACGGGGGGTTCGTGCACACAGCCCAGCTTGGAGCGAACGACCTACACCG<br>AACTGAGATACCTACAGCGTGAGCTATGAGAAAGCGCCACGCTTCCCGAA<br>GGGAGAAAGGCGGACAGGTATCCGGTAAGCGGCAGGGTCGGAACAGGA<br>GAGCGCACGAGGGAGCTTCCAGGGGGAAACGCCTGGTATCTTTATAGTCC<br>TGTCGGGTTTCGCCACCTCTGACTTGAGCGTCGATTTTTGTGATGCTCGTC<br>AGGGGGGCGGAGCCTATGGAAAAACGCCAGCAACGCGGCCTTTTTACGG<br>TTCCTGGCCTTTTGCTGGCCTTTTGCTCACATGTTCTTTCCTGCGTTATCCCC<br>TGATTCTGTGGATAACCGTATTACCGCCTTTGAGTGAGCTGATACCGCTCG<br>CCGCAGCCGAACGACCGAGCGCAGCGAGTCAGTGAGCGAGGAAGCGGA<br>AGAGCGCCTGATGCGGTATTTTCTCCTTACGCATCTGTGCGGTATTTCACA<br>CCGCAATGGTGCACTCTCAGTACAATCTGCTCTGATGCCGCATAGTTAAGC<br>CAGTATACACTCCGCTATCGCTACGTGACTGGGTCATGGCTGCGCCCCGAC<br>ACCCGCCAACACCCGCTGACGCGCCCTGACGGGCTTGTCTGCTCCCGGCAT<br>CCGCTTACAGACAAGCTGTGACCGTCTCCGGGAGCTGCATGTGTCAGAGG<br>TTTTCACCGTCATCACCGAAACGCGCGAGGCAGCTGCGGTAAAGCTCATC<br>AGCGTGGTCGTGAAGCGATTCACAGATGTCTGCCTGTTCATCCGCGTCCA<br>GCTCGTTGAGTTTCTCCAGAAGCGTTAATGTCTGGCTTCTGATAAAGCGGG<br>CCATGTTAAGGGCGGTTTTTTCCTGTTTGGTCACTGATGCCTCCGTGTAAG<br>GGGGATTTCTGTTCATGGGGGTAATGATACCGATGAAACGAGAGAGGAT

	GCTCACGATACGGGTTACTGATGATGAACATGCCCGGTTACTGGAACGTT<br>GTGAGGGTAAACAACTGGCGGTATGGATGCGGCGGGACCAGAGAAAAAT<br>CACTCAGGGTCAATGCCAGCGCTTCGTTAATACAGATGTAGGTGTTCCACA<br>GGGTAGCCAGCAGCATCCTGCGATGCAGATCCGGAACATAATGGTGCAG<br>GGCGCTGACTTCCGCGTTTCCAGACTTTACGAAACACGGAAACCGAAGAC<br>CATTCATGTTGTTGCTCAGGTCGCAGACGTTTTGCAGCAGCAGTCGCTTCA<br>CGTTCGCTCGCGTATCGGTGATTCATTCTGCTAACCAGTAAGGCAACCCCG<br>CCAGCCTAGCCGGGTCCTCAACGACAGGAGCACGATCATGCGCACCCGTG<br>GGGCCGCCATGCCGGCGATAATGGCCTGCTTCTCGCCGAAACGTTTGGTG<br>GCGGGACCAGTGACGAAGGCTTGAGCGAGGGCGTGCAAGATTCCGAATA<br>CCGCAAGCGACAGGCCGATCATCGTCGCGCTCCAGCGAAAGCGGTCCTCG<br>CCGAAAATGACCCAGAGCGCTGCCGGCACCTGTCCTACGAGTTGCATGAT<br>AAAGAAGACAGTCATAAGTGCGGCGACGATAGTCATGCCCCGCGCCCACC<br>GGAAGGAGCTGACTGGGTTGAAGGCTCTCAAGGGCATCGGTCGAGATCC<br>CGGTGCCTAATGAGTGAGCTAACTTACATTAATTGCGTTGCGCTCACTGCC<br>CGCTTTCCAGTCGGGAAACCTGTCGTGCCAGCTGCATTAATGAATCGGCCA<br>ACGCGCGGGGAGAGGCGGTTTGCGTATTGGGCGCCAGGGTGGTTTTTCTT<br>TTCACCAGTGAGACGGGCAACAGCTGATTGCCCTTCACCGCCTGGCCCTG<br>AGAGAGTTGCAGCAAGCGGTCCACGCTGGTTTGCCCCAGCAGGCGAAAAT<br>CCTGTTTGATGGTGGTTAACGGCGGGATATAACATGAGCTGTCTTCGGTAT<br>CGTCGTATCCCACTACCGAGATATCCGCACCAACGCGCAGCCCGGACTCG<br>GTAATGGCGCGCATTGCGCCCAGCGCCATCTGATCGTTGGCAACCAGCAT<br>CGCAGTGGGAACGATGCCCTCATTCAGCATTTGCATGGTTTGTTGAAAACC<br>GGACATGGCACTCCAGTCGCCTTCCCGTTCCGCTATCGGCTGAATTTGATT<br>GCGAGTGAGATATTTATGCCAGCCAGCCAGACGCAGACGCGCCGAGACA<br>GAACTTAATGGGCCCGCTAACAGCGCGATTTGCTGGTGACCCAATGCGAC<br>CAGATGCTCCACGCCCAGTCGCGTACCGTCTTCATGGGAGAAAATAATACT<br>GTTGATGGGTGTCTGGTCAGAGACATCAAGAAATAACGCCGGAACATTAG<br>TGCAGGCAGCTTCCACAGCAATGGCATCCTGGTCATCCAGCGGATAGTTA<br>ATGATCAGCCCACTGACGCGTTGCGCGAGAAGATTGTGCACCGCCGCTTT<br>ACAGGCTTCGACGCCGCTTCGTTCTACCATCGACACCACCACGCTGGCACC<br>CAGTTGATCGGCGCGAGATTTAATCGCCGCGACAATTTGCGACGGCGCGT<br>GCAGGGCCAGACTGGAGGTGGCAACGCCAATCAGCAACGACTGTTTGCCC<br>GCCAGTTGTTGTGCCACGCGGTTGGGAATGTAATTCAGCTCCGCCATCGCC<br>GCTTCCACTTTTTCCCGCGTTTTCGCAGAAACGTGGCTGGCCTGGTTCACC<br>ACGCGGGAAACGGTCTGATAAGAGACACCGGCATACTCTGCGACATCGTA<br>TAACGTTACTGGTTTCACATTCACCACCCTGAATTGACTCTCTTCCGGGCGC<br>TATCATGCCATACCGCGAAAGGTTTTGCGCCATTCGATGGTGTCCGGGATC<br>TCGACGCTCTCCCTTATGCGACTCCTGCATTAGGAAGCAGCCCAGTAGTAG<br>GTTGAGGCCGTTGAGCACCGCCGCCGCAAGGAATGGTGCATGCAAGGAG<br>ATGGCGCCCAACAGTCCCCCGGCCACGGGGCCTGCCACCATACCCACGCC<br>GAAACAAGCGCTCATGAGCCCGAAGTGGCGAGCCCGATCTTCCCCATCGG<br>TGATGTCGGCGATATAGGCGCCAGCAACCGCACCTGTGGCGCCGGTGATG<br>CCGGCCACGATGCGTCCGGCGTAGAGGATCGAGATCTCGATCCCGCGAAA<br>TTAATACGACTCACTATAGGGGAATTGTGAGCGGATAACAATTCCCCTCTA<br>GAAATAATTTTGTTTAACTTTAAGAAGGAGATATACCATGGGCAGCAGCC<br>ATCATCATCATCATCACAGCAGCGGCCTGGTGCCGCGCGGCAGCCATATG

JRF_A12-13_R	CAGGCCGCTGCTGTG
JRF_A12-13_F	CTCGAGCACCACCACCAC
JRF_AB4_E581A_GB	GGAAGAAGCAACAGCGGATCTCTGGCATGATATTCCTGCCGCGGCACGCGCGGGAGCCTCTTTGCCTTCGCCGGATCTTATGGCTGTAGGGCCAGATTTAGCGCGTGCTACTTGGCTCTTATCTGAGCTTCATGATGATTGTTCATCGGTCTATGGCGGTCTTgccCGTCGTGCCCATTCGGATCCTGATGGTCTCTGGGATGGATTTCTCCGCACGGTTAGTGCACATTTACCACCAGGCGGTATGGCGACGGTAGTGAGTCGCCGCCGTTCTGCGCACGTGACTCCAGAGCTTCCAGGTTTATCGG
JRF_AB4_E581_R	GGCAGGAATATCATGCCAGAGATC
JRF_AB4_E581_F	GCGCACGTGACTCCAG

As with our previous studies, the plasmids will be deposited at Addgene.

Recent reorganization and consolidation of databases have removed many of the original entries for the *amm* BGC. We therefore list below the amino acid sequences for all proteins.

Amm3:
MHGTHGRSDGTGGPGGGFAEEFRSFVASLQADEARTLRRWLDEAPDAPAPGPAAGPDG
GAPPAPDLEALFRDGESAPRDAGMDPTLRPRSDDPRAVRRIGVIGGGTAGYLTALALRAK
RPWLEVTLVESSGIPIIGVGEATTPSMVPFLHHYLGIDAQELYEAVQPTWKLGIRFDWGA
RPEGFMAPFDWGSNSIGVLGSLATRDSINSFTLQSLLMEQDKVPVFDLGGGRHMSLMKR
LPFAYHLDNQRFVRFLTELARKRGVGHLDARIERVVLDEETGWVDHLETDDGRRVDFDL
YVDCSGFRSLLLGQAMGSPEVSFADSLFTDRAVTAVVPHDGTIKPYTRATTMDAGWCW
TIPTRDDDHVGYVHSSRFISVEEATAELRRLYPRAHTFRTVHFRSGRHQEAWRGNVMAIG
NSYAFVEPLESSGLMMITLGILSLVSSLPGSWSQPCPRQVVNAALAKKWDEIRWFLALHY
RFNTRKDTPFWRTCREEADISGLEPLLEVFSGGAPLRFRDPVVRSFLESTAPTFYGLEGVD
CLLLGQGHPTRLLPMTEPVQAWRQREEAAQVLVSHAVDAATALRSFDRDARLNTQLLE
NEDSWVVRTGTDRLVSADPDAPGAVRARGVADADAPGDVSGGTSGDVSDGVSGRAEG
REDGDGRGGGDGRVGGGGDGTAAQADAAAGSAGGGERAGGVR
AmmA: VSETQVTETDNPAEEPAEIAAESDDLADLDDIEFDLDEVESKIAPLALALKESR

AmmC1:
MVNRPSDPRISSLLDGARTPLPGGAGTVRVPVDTGPAASYAGLRAALARLRAEGADFGG
LISRHGPEWAELFPADAPGEPPLAETALAPSERRLHRESEQNYRVLAVAASALCTGAEQT
GRPLELTGVGGTDLASLRGFMRAHEFARTRPGVRILLADPTAVRAAVLPEADYRAERAL
CLRRMGVGAAPADLRPVLPTRPEAGPGLGDGGTADARLYADAFGDGTAFDRLAAVLAG
CRRGFFTGNWEAMAALAATGGTLLDGFPDSRVADLLAAAREQDQQAEAIEFEPGILRTT
DDVRAFLAKVLGVQATFRGDQDRALAHFRAMRAGERLSPEVRAQSHLYAALTLTKRLG
SVDEAVAEVEEGFTAVPHREGEGNGVRRERGWLHNLRALTHFARRELREAFGHEKQAL
ACIEGLDDPSSIHLRVNLFSNVSVLQEKAGRHQQAARTWSRFKEAEGSANATFVKHHAY
RAGGLALLCGDRDTAREDLAKSIACAAEFNDDFHEAEIRTEVGTLLLADGDRAGAAAHF
ERAVALTSRIGDPYRMALAQLGRARAGGADPDPSVAALARLSTTHPARGAALADAAERG
GDAADGLLPAVRTKLNRPFDLINFQE

AmmB1:
MAATSGEGHWRLYPRLLLRRAGFDIGLLTGLTDPEVVGAAAGYRERAAAFERQREQCL
AGLRAEVDAAARDGDRSLLRSLSKARSRIGRRGPLDGRLSTVNDAATAYERARRERED A
WEVLRDALDAESADRPRRIRAVLDDDRVRDAVLQLAPSYAAEVERWSAGPRDRPANAK
TRAFFRRTYLYCQRLGAKNETTSFFGPLIHGTVAADGEGITLGPETPSGITDTEAFLAFWA
VGELARVMAEEPATAAGLPLTWIAACRREDGALVLPDGRRVGARGALGRVAALVDGTRT
PRDIARDSGLELAEVTAAVERLRRAGAVHRHPEPPSTEPRPLDWLIALADERAATTAWPE
ALRDLRDRAGLYAGAVGPVKRRRALEDVEAAFAALTGSDPRREGGRMYADRLVVSLDS
QGDQGPVTVGAATAARWERELAPVLDVAAHYGELLQSAASRLCADLMREAGTRTMPY
DELIRRSRAAVQQGRDRDHTGPADAFAAELTRTVTDAVRRPHGAPARAVLAPADLAELR
SPRARPRFVSPDLMLETREGRPETLVLGELHPYVFAWGSQGLFDHDPASSRAAFSGRLDP
WGGPERIATVIRRRRHKGLVAHWFPGRFVEITARATDDRARTLPVTDLTVELADGVPVLR
APDGELVLYAGEDDHVHLRAFAAPTAVLPPVVVDGVMPRWGVGDVVAQRARWWVDPA
RLLGDGPPSATGAYRTVQHLRTELALPRFVFAHLAGEPKPVGVDLDSPLAVETLTALVSA
SAADRVALTEMRPAPDRLWLRRQGRPVTSEFRLALRWETV

AmmB2:
MSTPAWHVLPTVVVRSAGFPWRLVDRLGHRKSRRLAGELVALRTEAAELAATAAPTGRL
ARSTAARLRNLRPLPADAPLPAPWLERWNDLTGRLAHTAGLLAATVEEEAEEAAAALAD
TTADPRFLEAVACSSPPAYRDLRKGARGARLRRQAASYAQRFAAKCETMSFFGPVNYGRI
DPGAPEAARWTWDGYEACPDRRAFTAARVTDRIEEALLADPDVVAALVPRRKTVLRPA
APDAGARPATRLTAAADGLRTVERLGHDLGLDPAVAVDACRTALTRGLLTHDWQPPATVA
DPVGWLAHRLDERPGGLSPRLRELVRDMHRILADYPAADAQTKVALQGRLTALAAGAV
PEPEPGRAGGTGRTAAGGPGRADPAGDLAGGPASGADRGASHGGGPAAVTSPGGPASAA
VAAEASAAEPRAAAVVSPSSAAEPRAAAVVSPSSPAEPRAAAVAAGSSGTGPRTAADPAA
TSHAGTGTGTGTDADAGAGTAPGAPRSGAAAPARPRDTGGDGTRTRFYNDRVIIHEAAR
GTLDLTVGGPLAADLTTAVAPVLDLLAHDAELTRAAANRAVAARLGPGRTPLLAAMRAC
ADLPFIRSDALTRRLAELLADPGTDTGAAALDLTGRLVPPPPSGPPVLCSVDVMIQTADL
AGYRPGVTPLILGDIHDAALLTPWALQFHPRAAALRAERDAAVLDTLGDQRAVTVVARR
STGLPPLDFPGLVLELGGTSDRDDAPRIGLDQLTVVSDGERAELRAAGHDAPLLFHNGEL
DTAFHTALALPRIRRPRLPALDHIPRLHFGNAVVARRRWTVPAADIAAVGQARGDADRLT
AAAALCAARALPETFFASSANERKPVYVDTSSPALLDCFLRLADTADVLTLSEAAPDRAD
SWLRHGGQPVAAELRCVYLRRAA

AmmB3:
MTRVWTLGTVFVVRHAGMPFDWLESLGGSADLHEAAERLLDAEEELRAAAGSAFPRL
AAAVEECAPERLPGVPAPAREAARAWRAAAARYREAHRTADEAATKELRRLLERPEVAE
AVLLSNPDALRNMLRPLLAHDGPLTSRYRRARRQLYTYVQRFCAKNETVSFFGPMAYGE
VDPGLPTADGALLSTRLPRTRRVFVSHWAGRALLRAVTRDTALLPDLPFHRTRDGEPAWP
DGASPEDAAAARTVLTALTPDGTTLRGLVRILGTPARELARALRLLVAADAADVGLGGG
PFDLEPLTTLRDGLDRLPPSAARDRWIARIEGLEALRAALQAAGPDEREAAIGALERAFT
EATGEPARRAAGSTYADRAVFYEEGSSPFALRIGPALAARWEQRLRGVLELCTAHGHLTQ
RAAADAVRSALGTGARLDLERYAAAAAEAFPAPGSAFAVAHAPDYPAADRERHTTRLRE
LADGLRGDRYALVDLCPRASGAAGLADADLVVARVHHHLLVPGWLDTMHPEPRRFAAA
AADWVARQDGRVVGFDFGRRNKGYYRFPGRAVALRPASWTDAGDPPLRPRDLTVELGE
PPGPAAGSGHGYETGPGYEPGYGRGSASGSGTGVRLLDPDGREVTAYIPLNDFVKYAPF

AALSHPQVLHPAFTAPEAAPEAAPQVASDAARDAAPDDPPGGAPGTAHGGTAPVDAAH
GGTDPGALPEVAVDGVVLQRPRWRVDASRLRRSAPHARFLELRRLARRTGLRFLFCRAP
RERKPYLLDLASPLAADLAAHIGHREDVLTAEAMSPAPGDLWLRDEQGRRYTCELRMQF
TGREVAAP

Amm12:
VFAEHARRAAHVHRPDGTERSDVTQATGCCADVHDGRGHVTHRFSDGPPSTAVPLPGV
PVAASVTAAVRALPGRELYEPDDATAKTLRELAQDGGVELTLGAYHQHVAAGTDAAVVT
DERLVCTVEASARTPQGVPCSEVVPWAAFGDPAESAARVAAAIDALHARRRLPWREDVP
GGCDLVLLPGRAGAFFHELVGHPMEADVFASGTSFLAGRAGRRVAPEWLHVVDGDEPA
GHGWRAVVDDEGTPRRPAVLIDRGRVGEPLTDLATALLRGADPTGHGRRLDHRHPAIAR
MTHTEAGVAPGTAPSPPPGDWIAPFDLQLQTMNLATGSFVFRSHTPLHHTADGTVHRLPP
LDLYGEGPRVLAGLEPAGTESAGYGRATKGCGKLGQFPLVVTFANAGIRLPAGLVTLREA
RDG

Amm13:
MAELLTTASALLTPTPNGPRSADSLDALLLERDRAGRVVASTRPGTTHSGFGAGAGIGAT
RDPYGTTTAAVDVPAHDPYGPTGEAGGGPGGPTAAALALLRRAYATWNGLPAAPLQSAV
VRVRERRAPDGTRWRSAGVTARLETVGGPVLVGSGRTGPALPETAVPALPSGPLADTPPA
SWQTRPLLLAPPVAAQIVTAAWYALTSASGRARLERLTGARVLPGLGLVDTADEHPPHGT
DDAGHPAGDLPVVTAGTLCPLPRDPEDGLLAGRLVWDHDTGRCVPAPHTALVLTGPAAP
RPADALELVHCVEGLQRHHADGVLRLLCLARPVDAPDRWSRFLLRGKPLKLLQAVRGA
TGAPDRVHTDHTVTVPALLLPAGGALAEKRTGSLAHS

Amm14:
LRTHDYLLVGGGIVGACLAEELARTGASVLVLDAGPAPGHATRQAAGVAVPSLRYLGRP
ALYDWLCAGRDRLEHDIARLEPGHGAFSVARPIVRALKPADADAHAERLDRLPGTRWA
ALPELSSLAPGLKLPAERRYLLDPRGLMVDGGRYLEAVHRSCLSAGVDWRQDTEVRDLT
ESPEGVTAATPRGTFAADRAVLTAGAWAGGRLAPGLPVRPLRGQLVVLDTPAELPLIFSSA
LYLAPGIGGGVIVGATEEDAGFDERCTAGGIAGLLRFAASAAPALASAAPRELRAGLRPA
TTTGTPLIGRIPGRDRTYVAAGHAGHGLLSARLTAEGLAGALTADRWDPIPEEFCPSVTLT
AGGTR

Amm17:
MTTTEPEIVILSGSLGETSRTNRLAQWCARQCASRAHPTVFAGADLAFPFYQPHLADPAP
QTRPFLGALSRADGVVVISPAYHGTVSGLLKNALDYVNELADAPQPYLDGRPLGCVAIG
AGDQGAASTLQTLRTVGHALRSWPTPMGVGLSQTRAELTGDGEPVDEQARNQLRVMT
EQVITLARINARRRAARAAARVNDLPAAPVSAGGVR

AmmB4:
VTSTRTAHPTDPGRADAPAPGVDGAGSPVPDPDGARGSAPGAAAADEGAAPAPGADGA
GSPVPDSGGVGGSAPRAAAADEGAAPAPGAGRASGVAAAAEVPSPQSGRPPGGRGTAP
RPRHAADPGTTAPLVWSVPHGPGTLTIRLLTPGLLRTPGFPAHLLEPVTAPATTALAAEAD
GRATREEADRRAFLDDRWPALRATARADGARHPAWRALLRAHRLVTEGRLLDPGAEAA
LTAVGGPALTAWAGEWNARRADDAAFRDRAAAAVRAATADAYRATDRAVDDPRVRHAV
FVSNPDFLDTALTPTPLARYGTGPDGVPPRRARRALATAHRYLRRLTTRCETVSFFGPVLF

TGFDSGRPEPVLRGAPAEERVLVEASTWLVEALGEDLTARTPPGLRIARRHPLFRPLDDGR
GLERVVDGKRFRLDPAALALWCAADGGTALDALAAGLGLDPAARDRAVRALGPTLLLT
GHPLPATCLDPTHRLAAHDPAGPAAALAEALAAHARTPWPERTASYGTVRAMARDLAG
GERKAAGAHYADREALFEDRVSPHSERLTFGGPVVDGIRRALSAVLPLCRLAALAARED
ARDTVRRATGGVAVPFVRLARAELPPDTPRLDRLRAVLRDLVAAGTPDADGAVHLTAAA
VEEATADLWHDIPAAARAGASLPSPDLMAVGPDLARATWLLSELHDDCSSVYGGLERRA
HSDPDGLWDGFLRTVSAHLPPGGMATVVSRRRSAHVTPELPGLSVELSGLSANDRAETL
PIAEVEVAADGSALLARGGRRLLYPGDLHSPLHRAVSLPALVPVDVETGDRTPRLVIDGV
VYQRARWRVPLPPAAGGEPYDRWLAVQRWRLGHGLPRRVFLRHPEEPKPLYVDFADPL
AVAETAALPPATVLVGEMLPAPGELWWSTGDGAQCAEFRLGCVVWPAEEGR

Amm19:
MSHRDRCRAAVERRRSTLLELSHSLHAEPETAFDEHRSAAKIADLLEGEGFAVERGTGGL
ATALTATWGDGELVAGVLAEYDALPGIGHACGHNVIAAASVGAALALREVAADLNLTVR
LIGTPAEESGGGKVLLLERGAFDDLAFALLIHPSPDEICAPRTLAVTDLEVTYRGRAAHAS
FAPHQGVNAADALTVAQVAIGLARQHLEPHQMVHGITTHGGDAPNIVPDRTGAVYYLRA
RSAESLSRLEHRVRDCLRAGAVATGCTEEIRRAAPAYTELVSDVRLTAAYREAITALGRVP
LDEETEARLVTPASTDMGNVSQVLPAIQPSIAVDARGAVNHQPGFAEVCAGPSGDAAVLD
GAVALAWTAVAAASDPAHRVALLDGVRARRCGAVR

Amm20:
MCGIVGWIAFDEDLTRRRDVLERMVATMARRGPDAAGHWLRPRAALGHRRLSVIDLEG
GAQPMTAGTPAGDVAITYSGETYNFTELRAELERAGHRFRTRSDTEVVLRAYLEWGEGL
VDRLNGMYALAIWDERTERLLLVRDRLGVKPLYYATTPGGVVFGSEPKALLANPHVPRR
VGADGLREIFGLVSTPGTAVWDGMAEVRPGHYAVVTRDGLREHCYWRLEARPHTDDRD
ATVERVGELLTDIVRRQLVSDVPRCSLLSGGLDSSLVTALAAAELGRVPGAGPVRSFDVD
FAHQEEGFRPDNMRGTPDSPYAREVAAHVGSDHRSVVLDPAALLDPGVRADTVLARDV
PLGWGELDTSLYLLFGGVRERSTVALSGESADEVFGGYGWFHDPELVAQPVFPWLLVTG
DHALGSMLHPELDAKTGVAEFRADCYADSLAATPVLPGEDPAERLMRQKLYMCLTRWL
RVMLDRKDRMSMAVGLEVRVPFCDHRLVEYVFGIPWSLKTFDGREKSPLRAAAAGLLP
SSVLERRKSPYPSTQDAAYTEGVQRWAGRLLDEPGSAVAELVDAAKLRRLVELPPRQLA
WGPRVALERVIDLDIWLRHYSPEIAV

Amm23:
VTEHATEVAPSDPWEQTRSTARPAPTDLRLRELVTGYRTSQVVYATVKLGLFDLLGARPR
TAAELAEAGGADPDALARFVRALTAERILQSTADGVLSLTPLGERLRSDAPGGLAAWVV
TSCEEQFFAWGHAVHTLRTGQSAFEKAHGVGFWEHLRSDAKAAESYDAGMAVSAGEA
CDLIVSAGGIDRARHVVDVGGGRGALARRLLDTFPHARVTVLDLPEVVERTRASFAPDP
VPERLGLVPGSFLDGVPAGGDVYVLSRVLADWDDAHAARILASCRRAMTPGGRLLIAE
GLTRDDAPAGARGLLDLHLLLLFGGRERTVTGLTALLSGAGFALVSVTGADNGGISLLTA
VAR

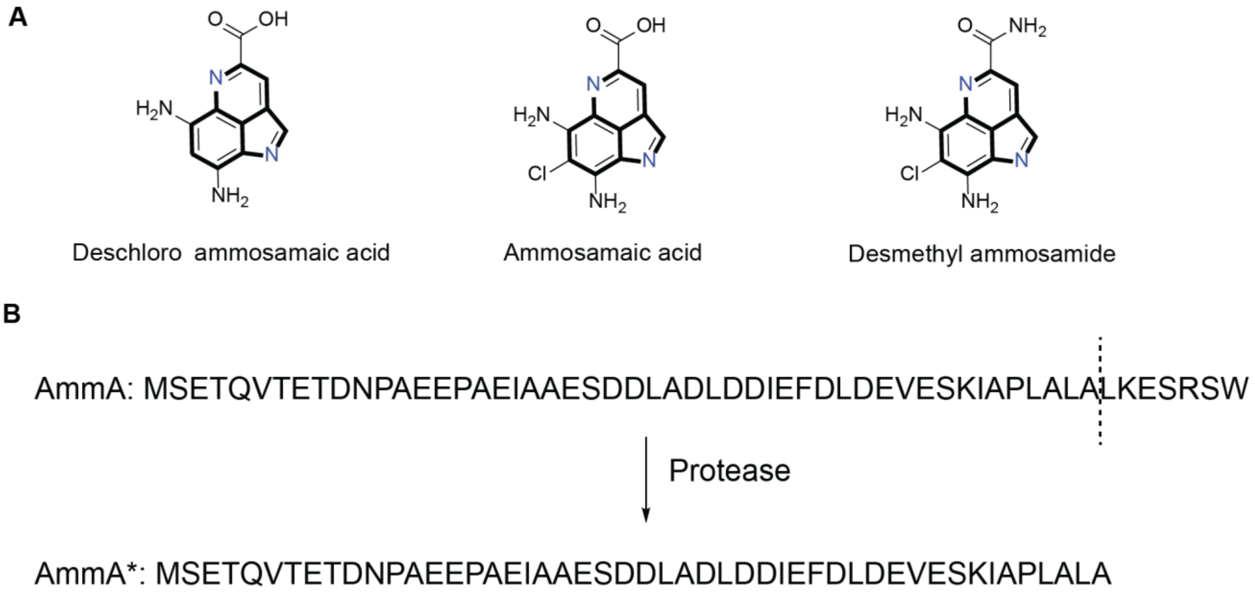

Figure S1. (A) Pyrroloiminoquinone metabolites proposed and identified from ammosamide-related BGCs. Ammosamaic acid was identified from a strain in which the gene for Amm3 (chlorinase) was disrupted and desmethyl ammosamide was identified from a strain in which the gene for Amm23 (methyltransferase) was disrupted.[5] Deschloro ammosamaic acid was proposed as an intermediate in the lymphostin BGC.[6] (B) Precursor peptide sequence AmmA and AmmA* obtained after protease processing.[7]

Table S2. ESI-MS/MS analysis of in vivo produced intermediate **7** digested with trypsin, corresponding to Figure 2C. Experimental and theoretical values represent $[M+H]^+$ ions.

Fragment Type	Fragmented Bond Number	Neutral Loss	Experimental m/z	Theoretical m/z	Mass Error (ppm)
a	1		86.1	86.1	5.6
a	2		157.1	157.1	6.1
y	1	-NH3	158.1	158.1	8.2
y	1		175.1	175.1	6.6
b	2		185.1	185.1	3.5
b	3		282.2	282.2	3.1
a	4		367.3	367.3	3.4
y	2	-NH3	388.2	388.2	3.8
b	4		395.3	395.3	4.4
y	2		405.2	405.2	2.8
a	5		438.3	438.3	1.0
y	3	-NH3	459.2	459.2	1.2
b	5		466.3	466.3	2.0
y	3		476.2	476.2	1.9
a	6		551.4	551.4	-1.5
y	4	-NH3	572.3	572.3	1.9
b	6		579.4	579.4	0.8
y	4		589.3	589.3	-0.2
y	5	-NH3	643.3	643.3	5.6
b	7		650.4	650.4	2.8
y	5		660.4	660.4	-3.0
a	8		852.5	852.5	1.3
y	7	-NH3	853.5	853.5	7.2
y	7		870.5	870.5	-3.2
b	8		880.5	880.5	3.0
M+H		-NH3	1037.6	1037.6	2.9
M+H			1054.6	1054.6	2.2

A

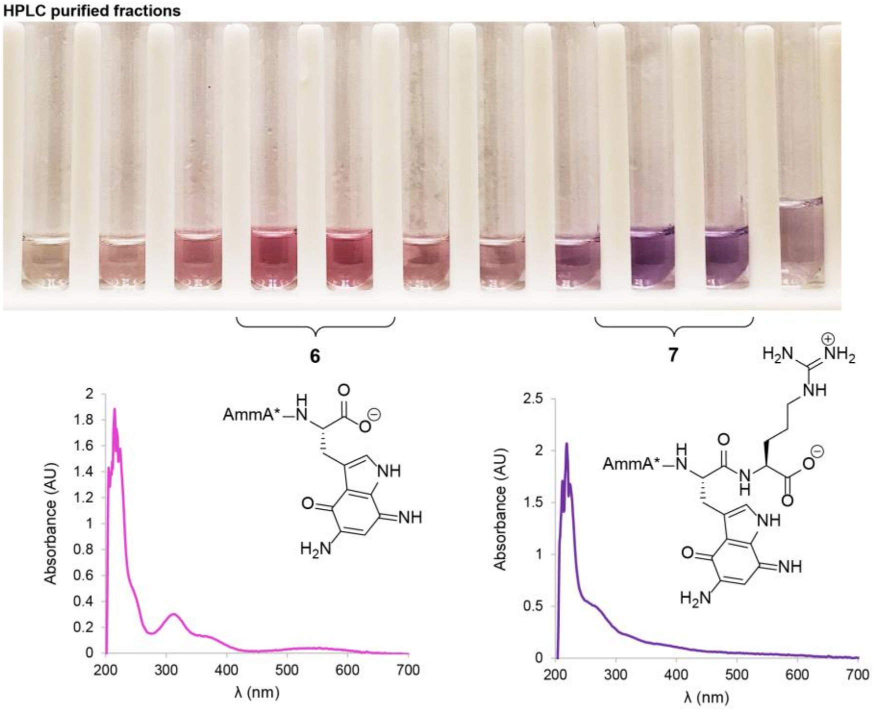
HPLC purified fractions
6
7
AmmA*
NH
H_2N
Absorbance (AU)
λ (nm)

B

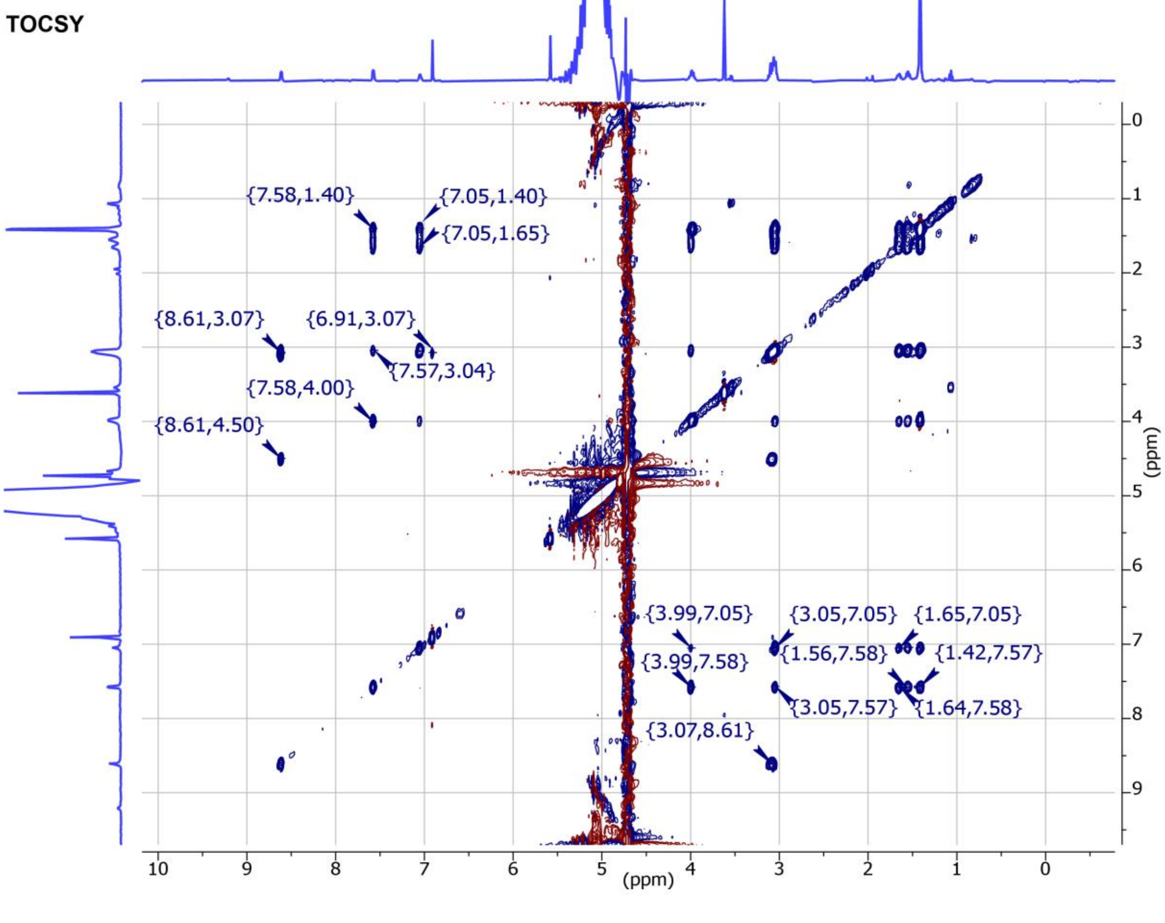
TOCSY
{7.58,1.40}
{7.05,1.40}
{7.05,1.65}
{8.61,3.07}
{6.91,3.07}
{7.57,3.04}
{7.58,4.00}
{8.61,4.50}
{3.99,7.05}
{3.05,7.05}
{1.65,7.05}
{1.56,7.58}
{1.42,7.57}
{3.99,7.58}
{3.05,7.57}
{1.64,7.58}
{3.07,8.61}
(ppm)

C

NOESY

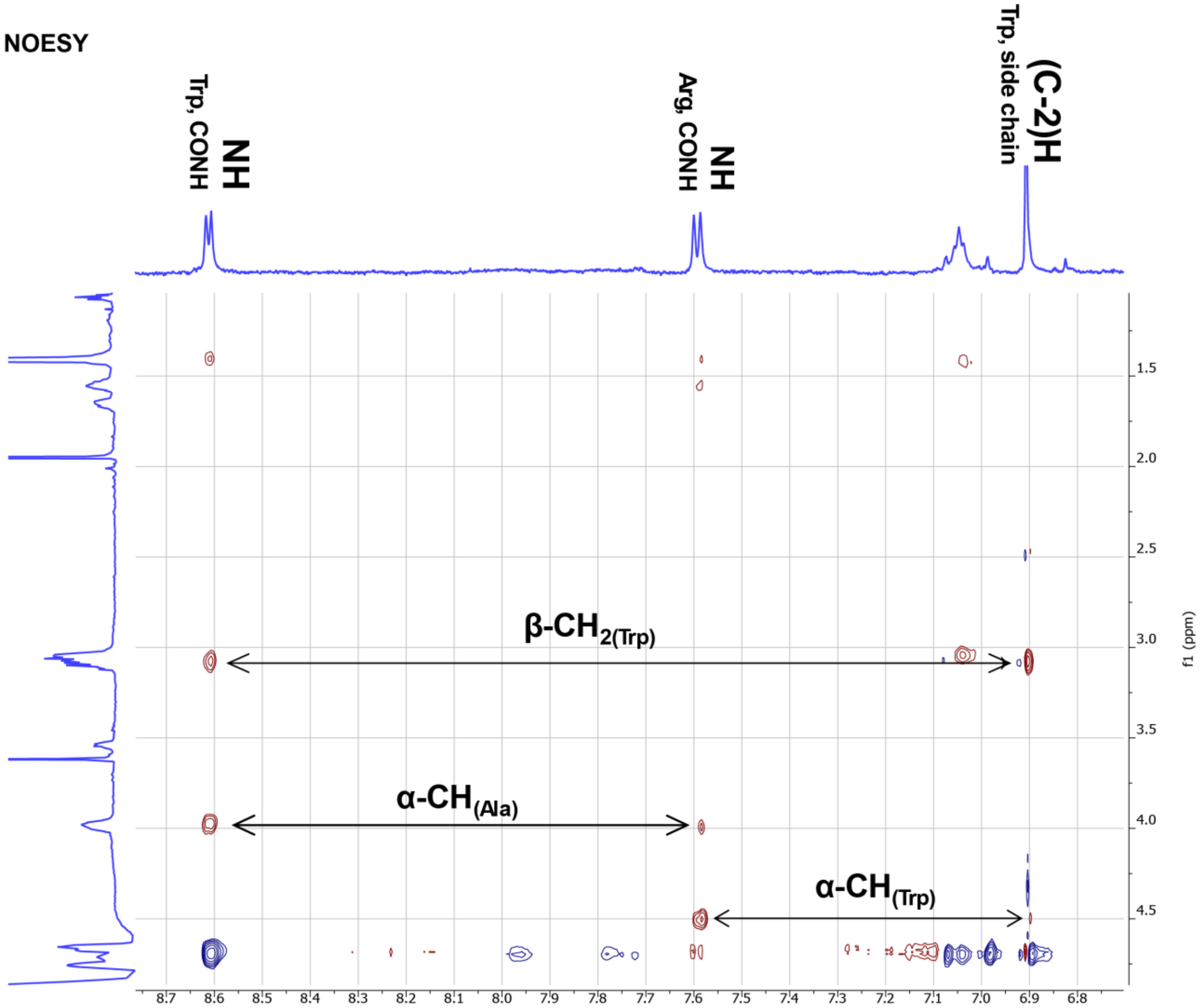

D

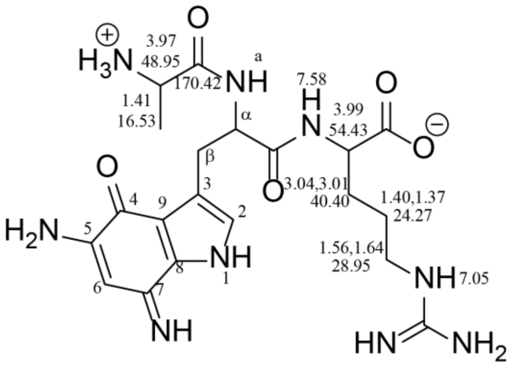

E

Trp	δ_H (ppm)	δ_C (ppm)
a	8.61	n.a.
α	4.5	n.o.
β	3.07	27.17
2	6.91	126.5
3	n.a.	121.92
4	n.a.	177.32
5	n.o.	n.o.
6	5.57	89.94
7	n.o.	n.o.
8	n.a.	119.62
9	n.a.	128.65

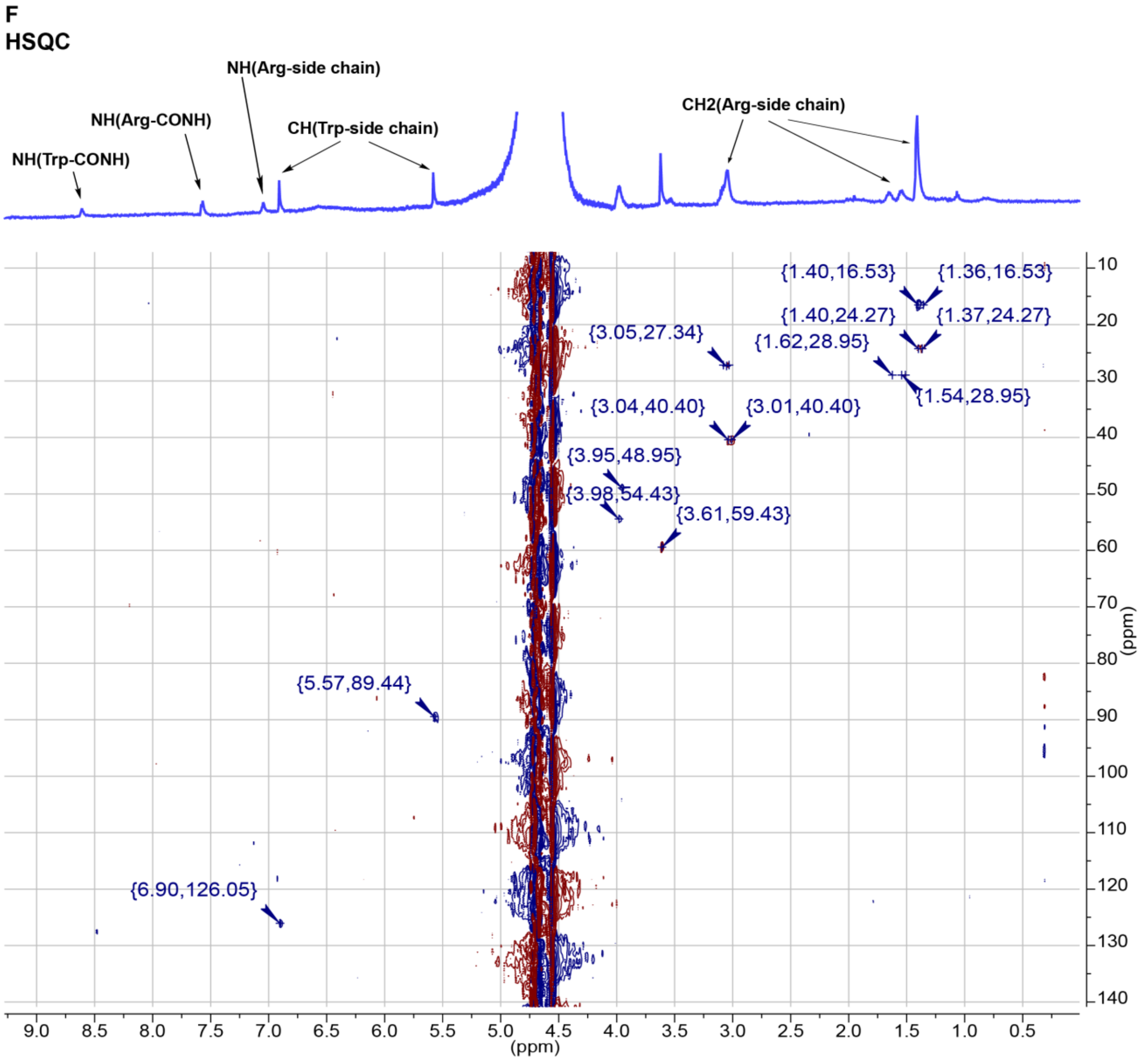

Figure S2. Purification and characterization of compound **7**. (A) Illustration of the change in color upon adding Arg to peptide **6**. (B) 2D NMR analysis (TOCSY) of a chymotrypsin fragment of intermediate **7** in 10% D_2O, using a D_2O matched Shigemi tube. (C) 2D NMR analysis (NOESY) of a chymotrypsin fragment of intermediate **7** in 10% D_2O, using a D_2O matched Shigemi tube. (D) Chemical shifts of the chymotrypsin fragment of **7**. (E) Observed chemical shifts and assignments for the modified Trp. n.a.=not applicable, n.o.=not observed. (F) Heteronuclear Single Quantum Coherence (HSQC) spectrum of a chymotrypsin fragment of intermediate **7** (panel D) in 10% D_2O, using a D_2O matched Shigemi tube.

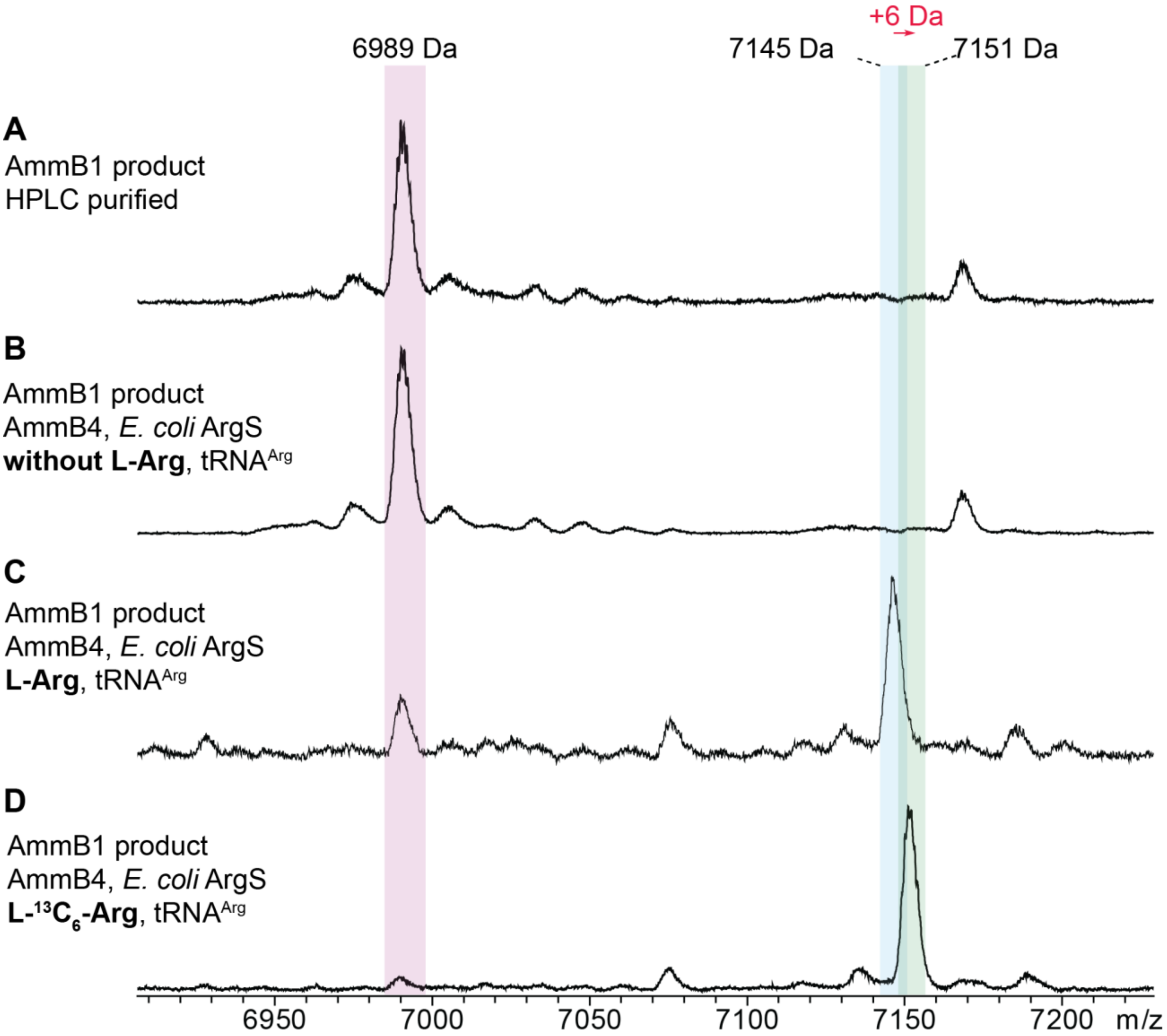

Figure S3. AmmB$_4$ reaction using purified tRNA^{Arg}, *E. coli* ArgRS, HPLC purified substrate (intermediate **6**) with and without L-Arg. (A) Intermediate **6** reference compound as purified by HPLC. Calculated average m/z for $[M+H]^+$ for **6**: 6990 Da, observed average m/z for $[M+H]^+$ for **6**: 6989 Da. (B) In vitro reaction using tRNA^{Arg}, *E. coli* ArgRS, AmmB$_4$, intermediate **6** but without L-Arg. (C) In vitro reaction using tRNA^{Arg}, *E. coli* ArgRS, AmmB$_4$, intermediate **6**, and L-Arg. Calculated average m/z for $[M+H]^+$ for **7**: 7146 Da, observed average m/z for $[M+H]^+$ for **7**: 7145 Da. (D) In vitro reaction using tRNA^{Arg}, *E. coli* ArgRS, AmmB$_4$, intermediate **6**, and L-$^{13}C_6$-Arg. Calculated average m/z for $[M+H]^+$ for **7**: 7152 Da, observed average m/z for $[M+H]^+$ for **7**: 7151 Da.

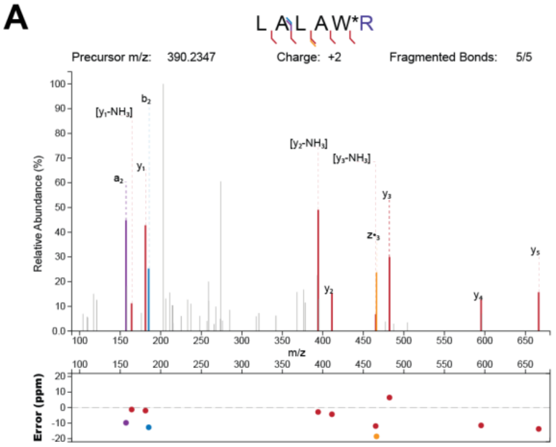

Fragment Type	Fragmented Bond Number	Neutral Loss	Experimental Mz	Theoretical Mz	Mass Error (ppm)
a	2		157.132	157.1335	-9.7742
y	1	-NH3	164.1126	164.1122	2.4249
y	1		181.139	181.1388	1.3738
b	2		185.1261	185.1285	-12.6964
y	2		206.1129	206.1132	-1.3042
y	2	-NH3	394.1921	394.1925	-1.0453
y	2		411.218	411.2191	-2.5805
y	3	-NH3	465.2248	465.2296	-10.3735
y	3		482.26	482.2562	7.9312
y	4		595.3341	595.3402	-10.3053
y	5		666.3689	666.3773	-12.6792

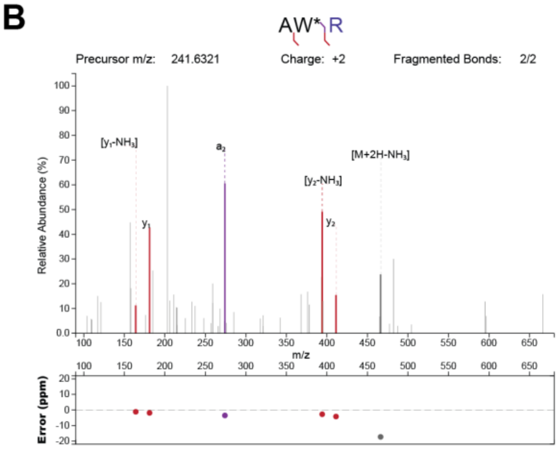

Fragment Type	Fragmented Bond Number	Neutral Loss	Experimental Mz	Theoretical Mz	Mass Error (ppm)
y	1	-NH3	164.1126	164.1122	2.4249
y	1		181.139	181.1388	1.3738
y	2		206.1129	206.1132	-1.3042
a	2		274.1289	274.1298	-3.2315
y	2	-NH3	394.1921	394.1925	-1.0453
y	2		411.218	411.2191	-2.5805
M+2H		-NH3	466.2295	466.2369	-15.8772

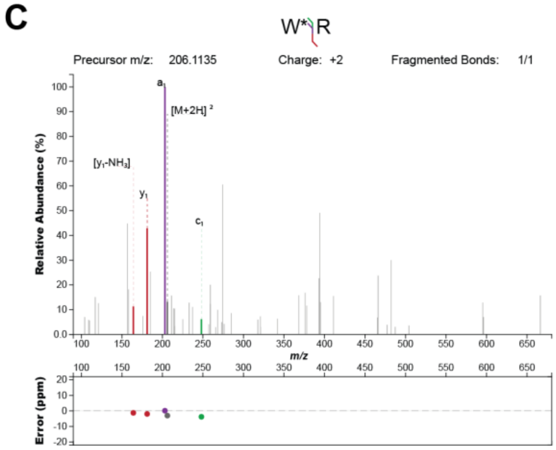

Fragment Type	Fragmented Bond Number	Neutral Loss	Experimental Mz	Theoretical Mz	Mass Error (ppm)
y	1	-NH3	164.1126	164.1122	2.4249
y	1		181.139	181.1388	1.3738
a	1		203.0928	203.0927	0.631
M+2H			206.1129	206.1132	-1.3042

Figure S4. ESI-MS/MS analysis of the $[M+2H]^{2+}$ ion for the C-terminal modified intermediate **7** with labeled L-$^{13}C_6$-Arg and digested with proalanase. (A) Fragmentation pattern for LALAW*R, where R is $^{13}C_6$-Arg. (B) Additional fragmentation of 390 Da ion produces AW*R, where R is also $^{13}C_6$-isotopically labeled. (C) Additional fragmentation of 206 Da ion produces W*R, where R is also $^{13}C_6$-isotopically labeled.

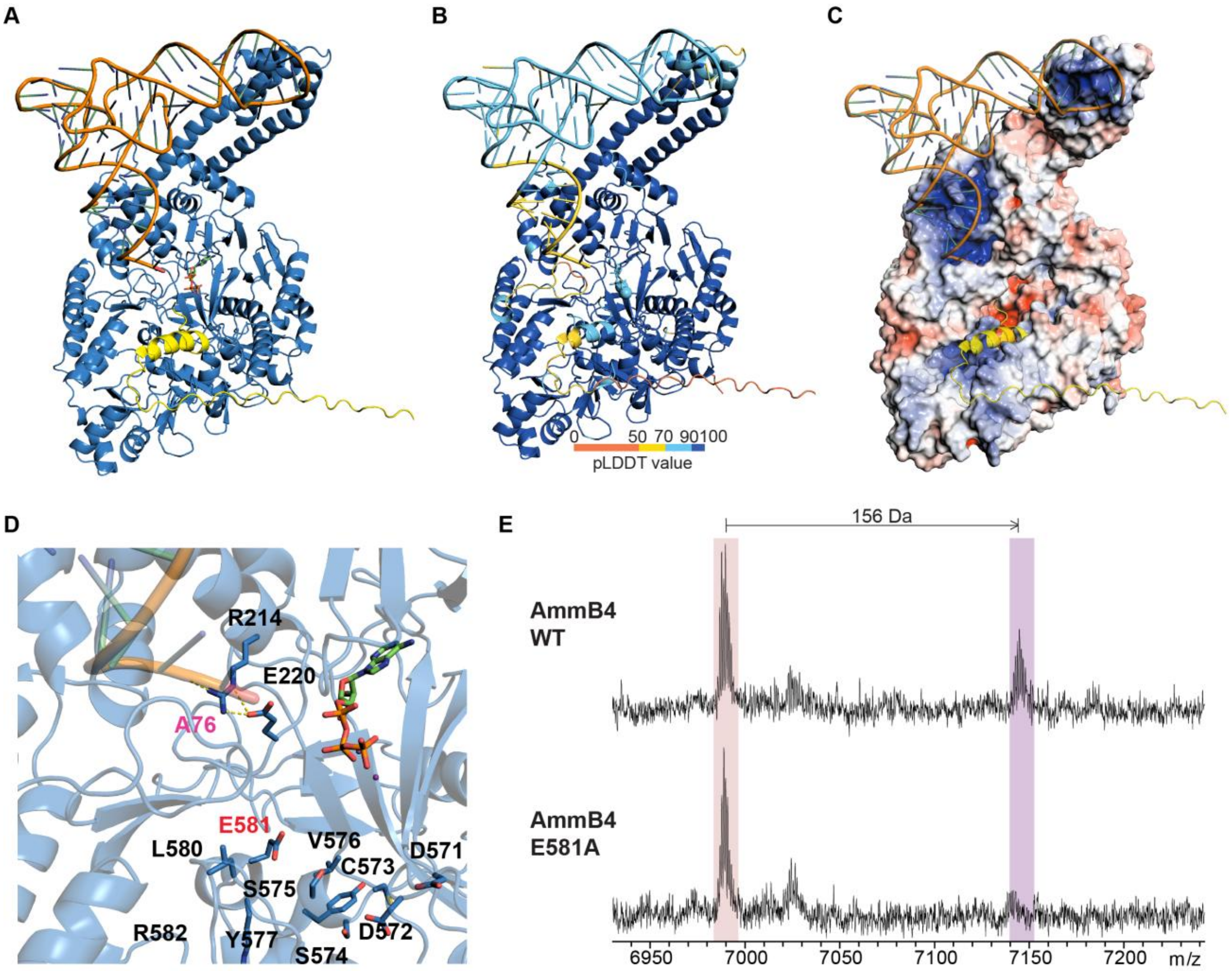

Figure S5. (A) AlphaFold3[8] predicted structure of $AmmB_4$ (in blue cartoon) in quaternary complex with $tRNA^{Arg}$ (in orange ribbon), AmmA*W (in yellow cartoon), ATP (in green sticks), and two Mg^{2+} ions (in purple spheres) visualized by PyMOL. (B) AlphaFold3 predicted structure of the quaternary complex colored by predicted local distance difference test (pLDDT) scores. (C) Electrostatic surface potential calculated for $AmmB_4$. Positive potential (blue color) overlaps with tRNA contacts as well as some parts of the peptide substrate (in yellow). Negative potential (red color) is observed in the enzyme active site near the C-terminus of the peptide substrate (yellow) and the A76 nucleotide that forms the 3' terminus of $tRNA^{Arg}$. (D) Zoom-in showing Glu581 located in a helix positioned between the 3'-CCA and ATP binding site. Glu581 can tentatively interact with the side chain of the Arg attached to the tRNA. Glu220 is conserved in all PEARLs and is unlikely to provide recognition of the Arg side chain. (E) MALDI-TOF MS data for the in vitro activity assay of wild-type $AmmB_4$ (WT, top panel) compared to the mutant $AmmB_4$-E581A (bottom panel) in the presence of ATP, Mg^{2+}, AmmA*W, and Arg-tRNA indicating that Glu581 is important for arginine recognition. The mutated enzyme $AmmB_4$ was not able to catalyze the addition of Arg.

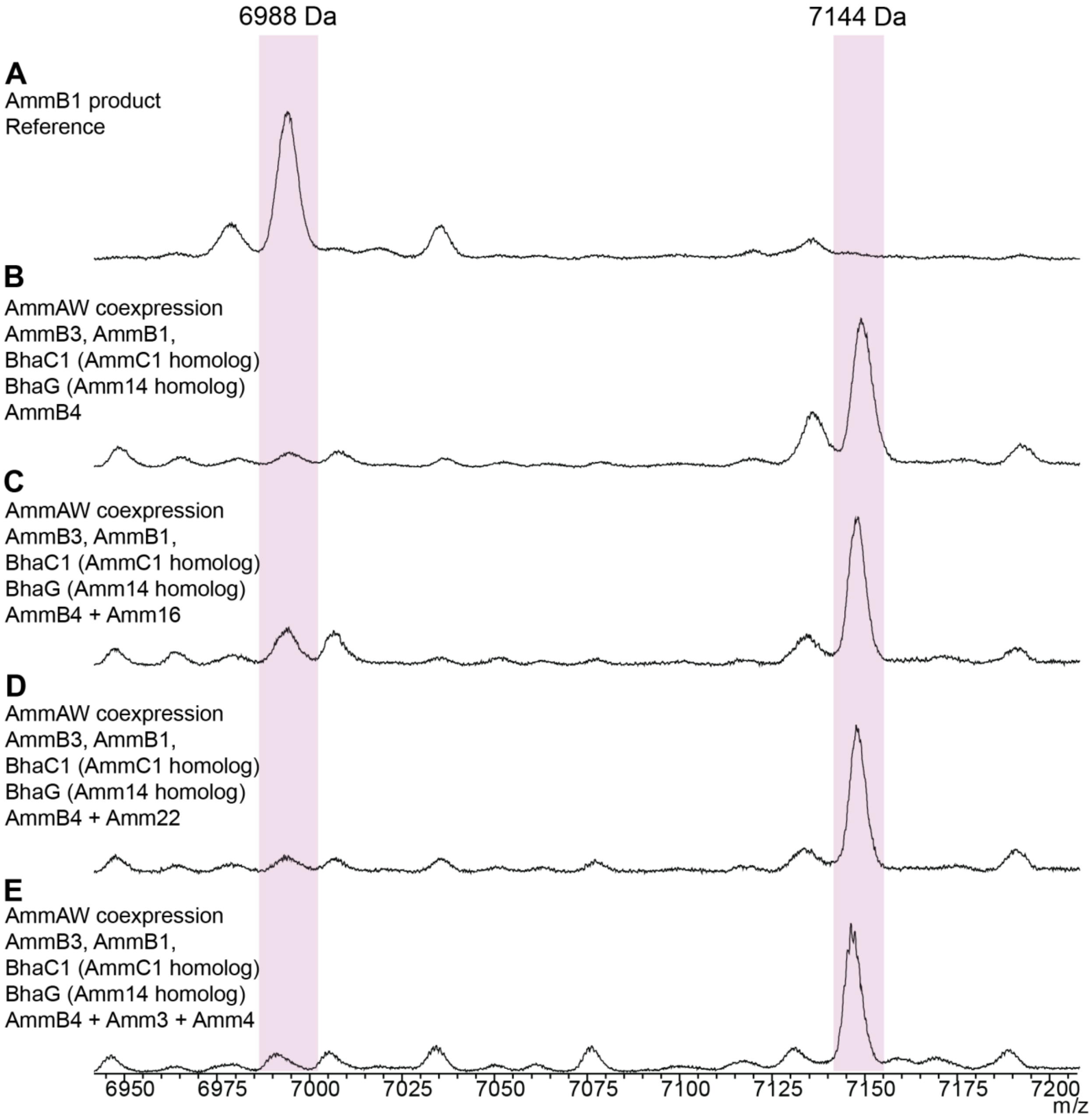

Figure S6. MALDI-TOF mass spectra of co-expressions with various enzymes (indicated in red font below) that did not lead to additional mass changes after modification by $AmmB_4$. (A) Peptide obtained after HPLC purification of the product from co-expression of AmmA*W, $AmmB_3$, $BhaC_1$ (AmmC1 homolog), BhaG (Amm14 homolog), and $AmmB_1$. (B) Product of co-expression of AmmA*W, $AmmB_3$, $BhaC_1$ ($AmmC_1$ homolog), BhaG (Amm14 homolog), $AmmB_1$, and $AmmB_4$. (C) Product of co-expression of AmmA*W, $AmmB_3$, $BhaC_1$ ($AmmC_1$ homolog), BhaG (Amm14 homolog), $AmmB_1$, $AmmB_4$, and **Amm16**. (D) Product of co-expression of AmmA*W, $AmmB_3$, $BhaC_1$ ($AmmC_1$ homolog), BhaG (Amm14 homolog), $AmmB_1$, $AmmB_4$, and **Amm22**. (E) Product of co-expression of AmmA*W, $AmmB_3$, $BhaC_1$ ($AmmC_1$ homolog), BhaG (Amm14 homolog), $AmmB_1$, $AmmB_4$, and **Amm3 and Amm4**.

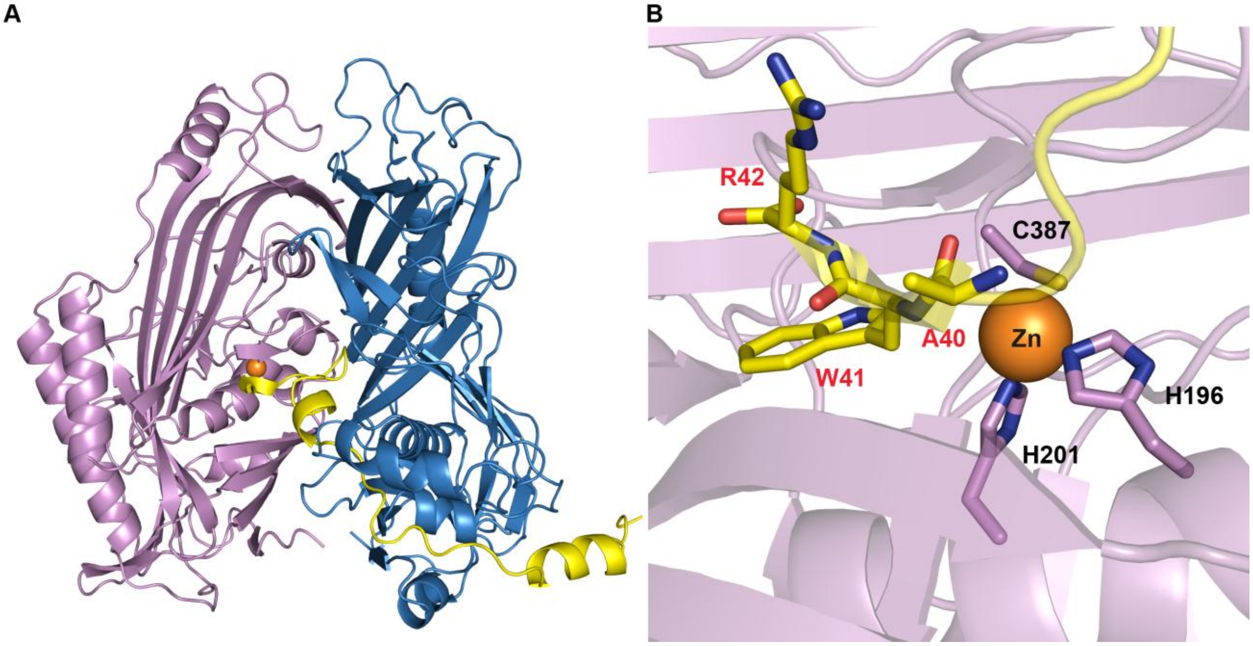

Figure S7. AlphaFold3 predicted structure of the Amm12/Amm13 complex with AmmA*WR and Zn^{2+}. (A) Complex of Amm12 (pink) and Amm13 (blue) with a putative interaction with the C-terminus of AmmA*WR (yellow) and Amm12 through a beta sheet interaction proximal to the Zn^{2+} ion (in orange). (B) Predicted Zn^{2+} binding site involves interactions with His196, His201, and Cys387 residues in Amm12. The amide carbonyl bond between Ala40 and Trp41 is observed near the Zn^{2+} ion as expected for C-terminal dipeptide cleavage.

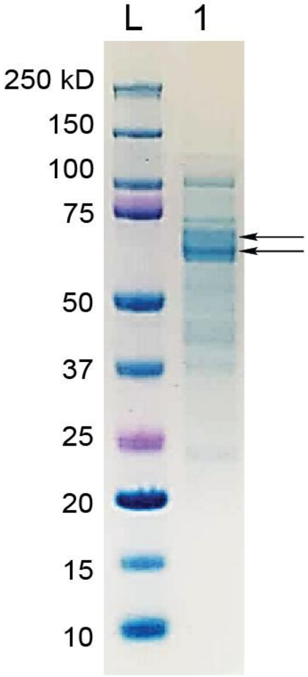

Figure S8. SDS-PAGE for the expression of His-tagged-Amm12 with untagged Amm13 after size exclusion chromatography.

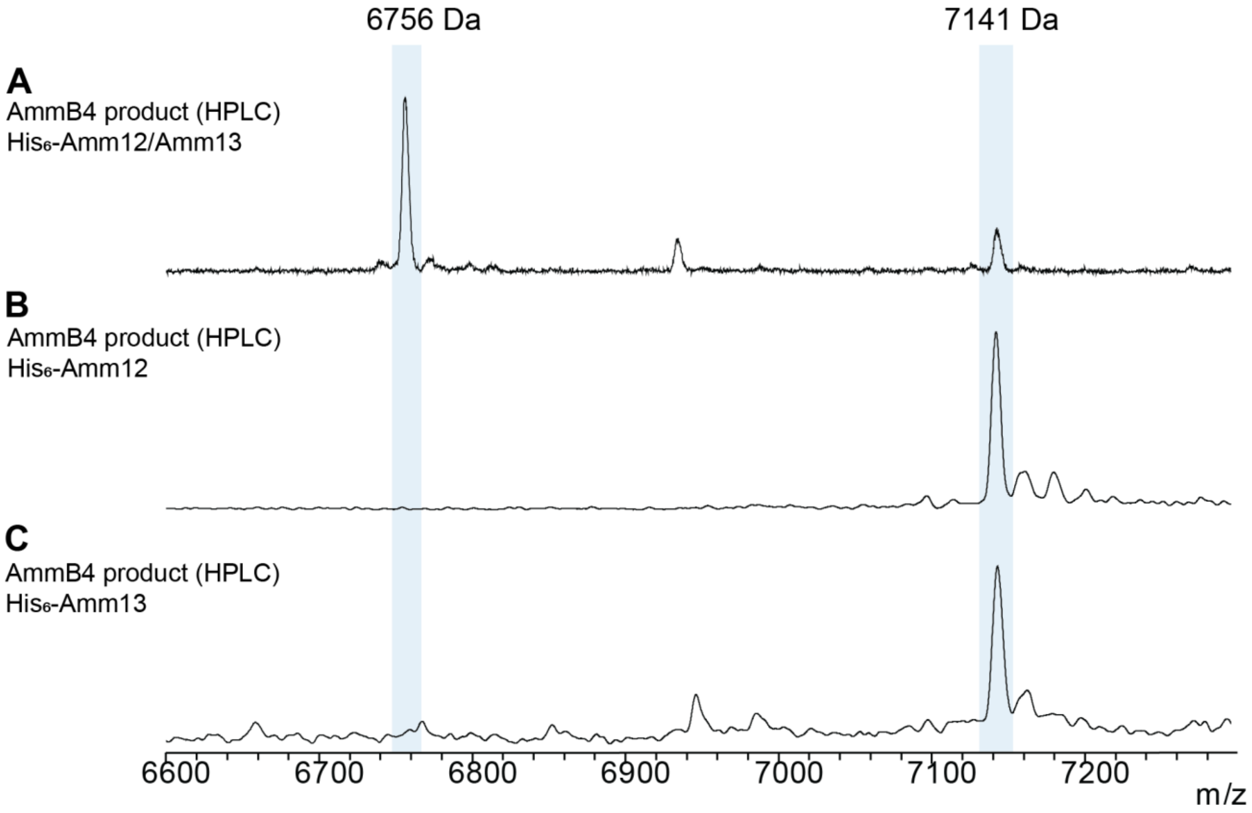

Figure S9. MALDI-TOF mass spectra of in vitro reactions of intermediate **7** with (A) His-tagged Amm12 and pulled down Amm13, (B) His-tagged Amm12, (C) and His-tagged Amm13.

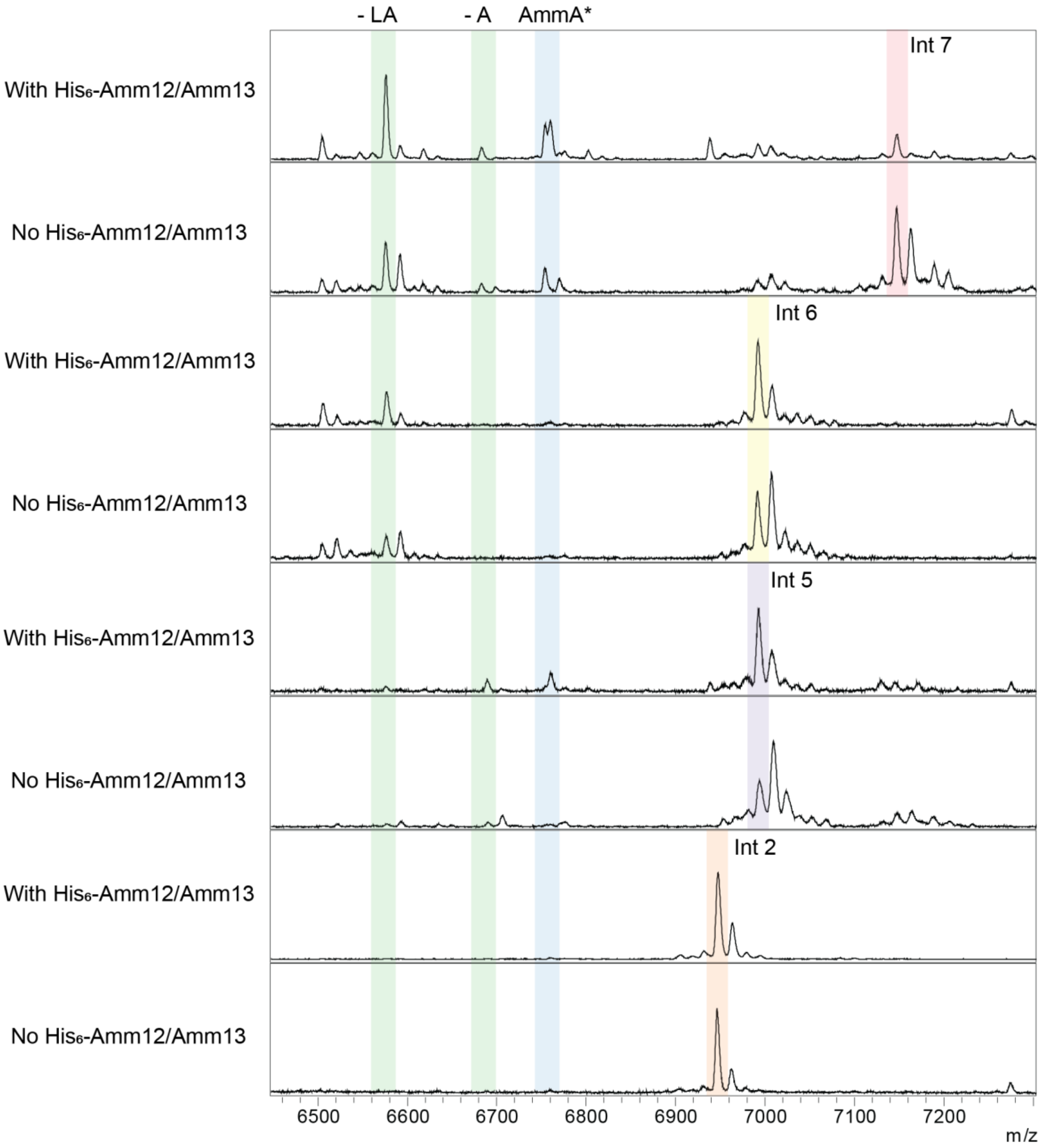

Figure S10. Substrate recognition analysis for Amm12/Amm13 activity using intermediates **2**, **5**, **6**, and **7**. While peptide **7** was mostly processed producing AmmA* with corresponding loss of dipeptide **8**, additional removal of the dipeptide Leu-Ala is also observed. All other intermediates that lack the C-terminal Arg residue are not recognized by Amm12/Amm13 for C-terminal proteolysis.

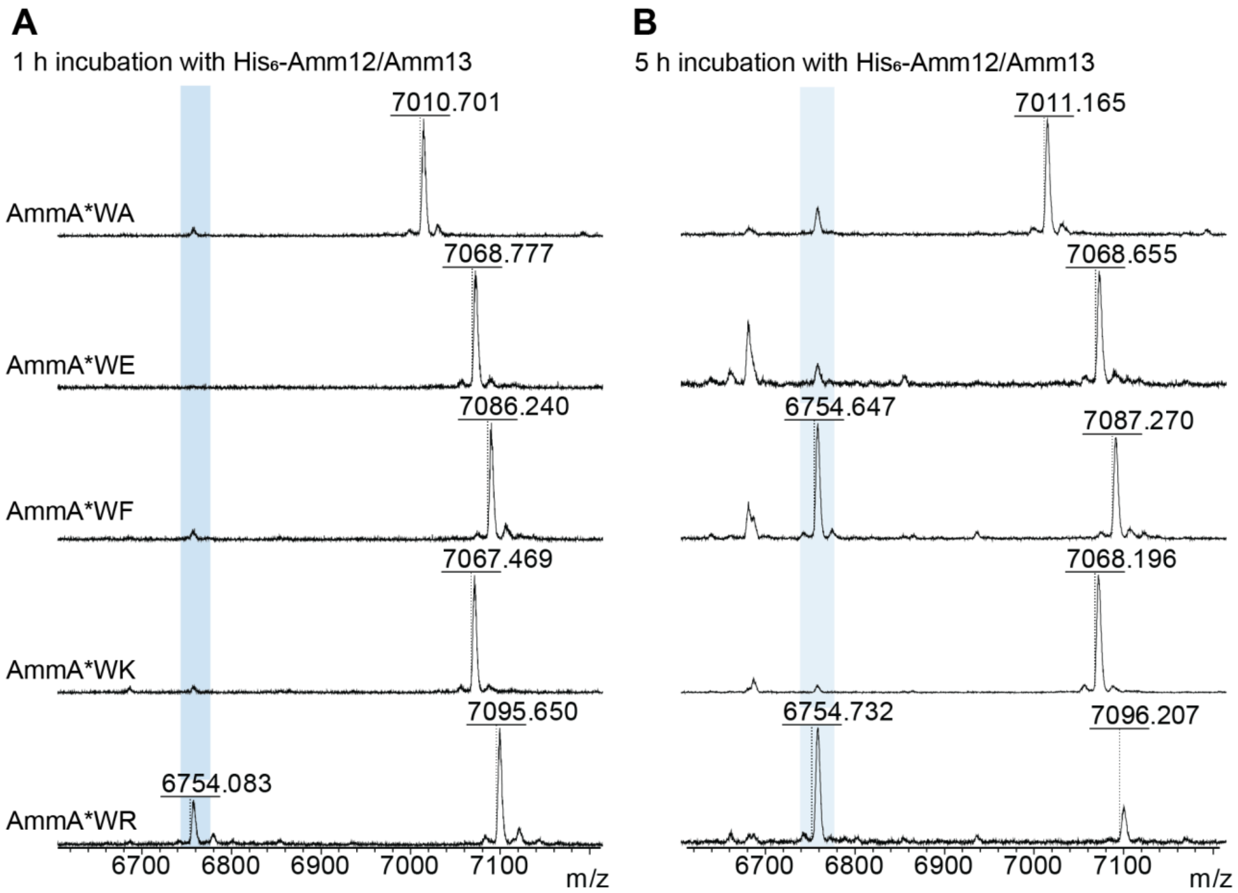

Figure S11. Amm12/Amm13 substrate scope. Several analogs of intermediate **7** (AmmA*WA, AmmA*WE, AmmA*WF, AmmA*WK, and AmmAW*R) were expressed and purified as His-tagged fusion constructs. All peptides lacked the posttranslational modifications at the Trp that are preent in **7**. (A) After 1 h incubation at 37 °C with Amm12/Amm13, only analog AmmA*WR was partially cleaved with removal or WR and formation of AmmA*. (B) After 5 h incubation, AmmA*WF was partially cleaved, AmmA*WR was almost fully processed, while AmmA*WA, AmmA*WE, and AmmA*WK were only slightly digested.

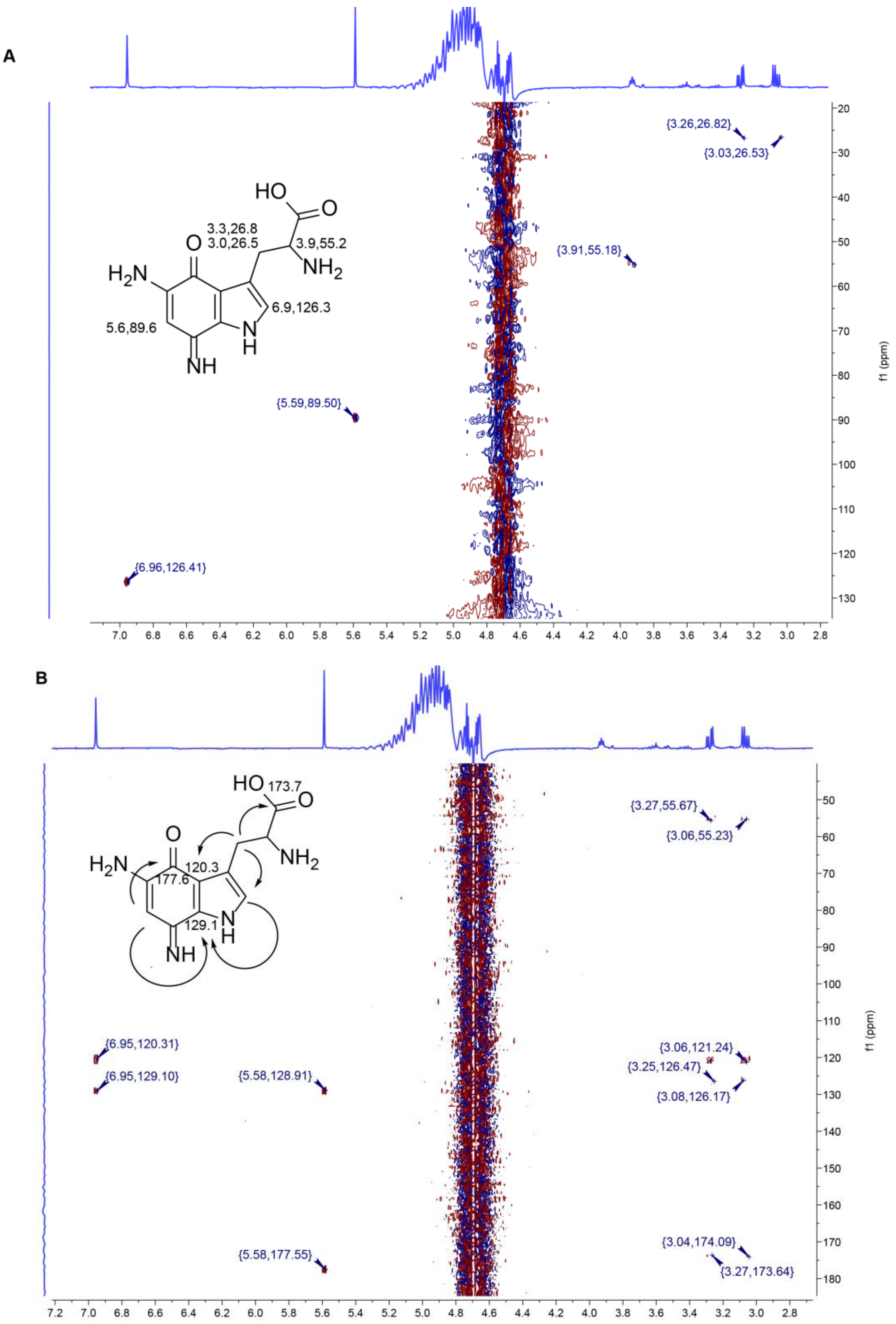

Figure S12. 2D NMR analysis of intermediate **9** in 90% H_2O and 10% D_2O, using a D_2O matched Shigemi tube. (A) Heteronuclear Single Quantum Coherence (HSQC) spectrum. (B) Heteronuclear Multiple Bond Correlation (HMBC) spectrum. Arrows indicate the correlation between protons and corresponding carbons three-bonds away.

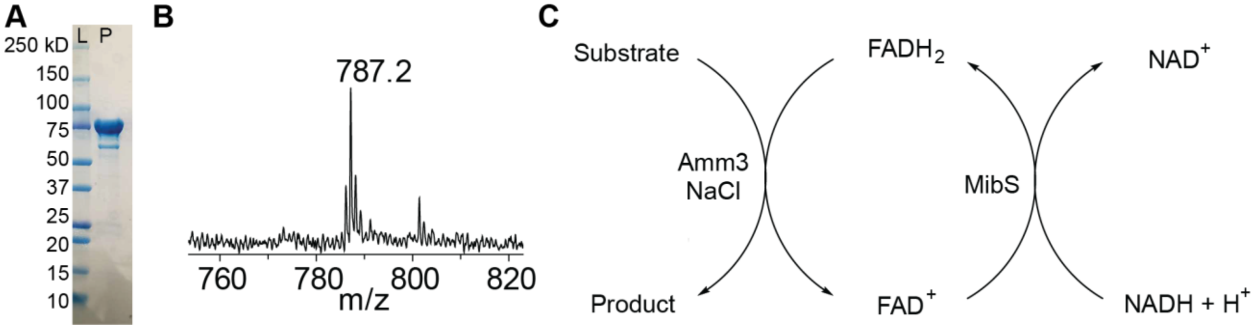

Figure S13. (A) SDS-PAGE gel obtained for His-tagged Amm3 coexpressed with chaperones as mentioned in the methods description. Molecular weight for His-tagged Amm3 is 74 kDa. (B) MALDI-TOF mass spectrum of the cofactor released after boiling an aliquot of purified Amm3. Expected mass for FAD: 785.2 Da, observed mass: 787.2 Da. (C) In vitro cascade reaction for Amm3 activity. MibS copurifies with bound FAD, which can be reduced using NADH. The reduced flavin, $FADH_2$, O_2 and Cl^- form HOCl, the activated form required for Amm3 chlorination of accepted substrates.

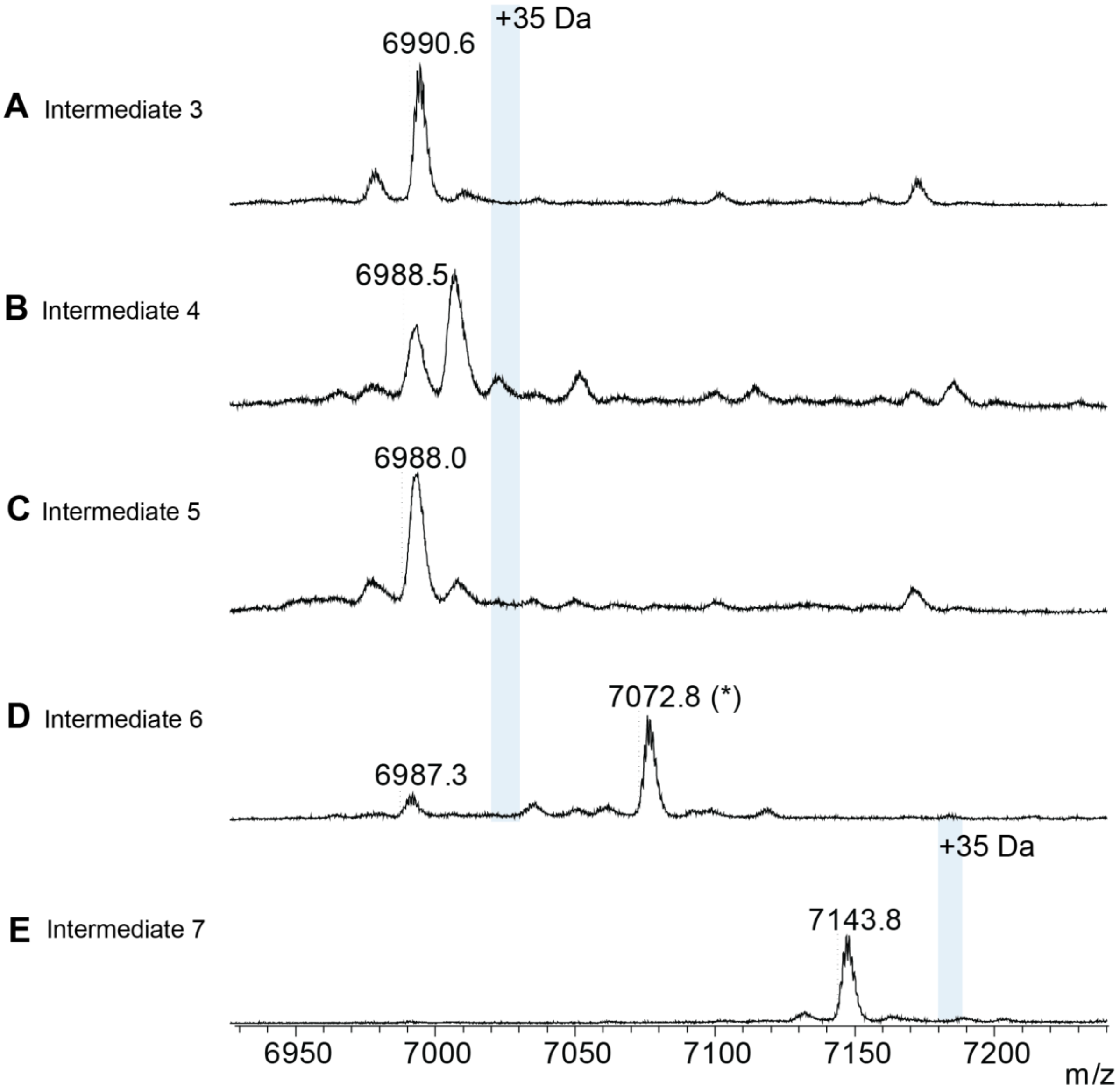

Figure S14. MALDI-TOF mass spectra of the products of various peptides incubated with Amm3 in vitro. * indicates diacetylated intermediate **6** produced due to further processing during *E. coli* expression.[1]

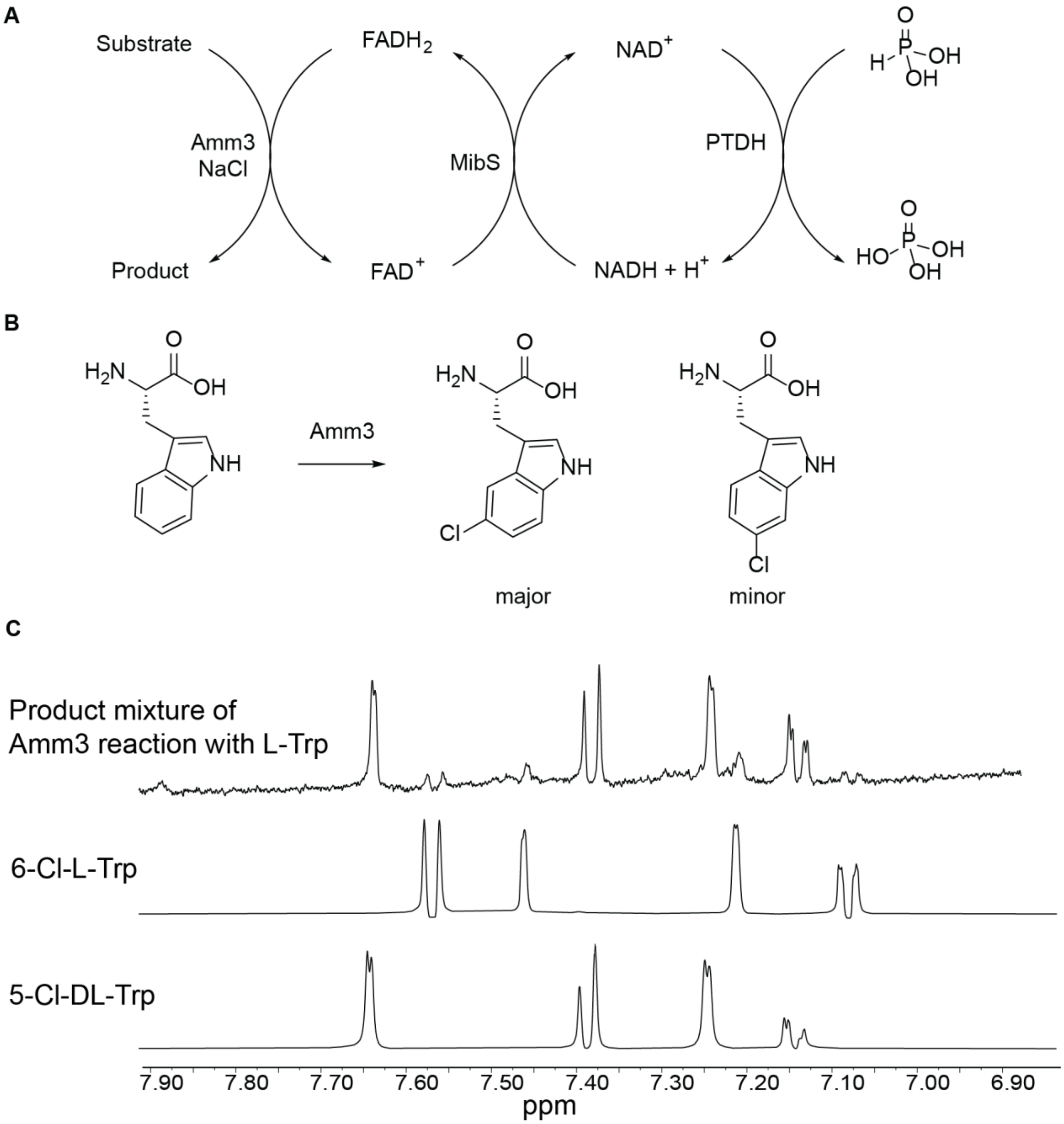

Figure S15. Reaction of Amm3 with Trp. (A) In vitro cascade reaction to recycle NADH as a cofactor was implemented by using phosphite dehydrogenase (PTDH) as a coupling enzyme.[4] H_2O_2 built up was decreased by using catalase. (B) Trp was accepted as a substrate for Amm3. Chlorination was observed mostly at C-5 of the indole, with minor C-6 chlorination. (C) Isolation of the chlorinated product of the Trp reaction with Amm3 by HPLC and analysis by ^{1}H NMR revealed the major product of the reaction is 5-Cl-Trp whereas minor amounts of 6-Cl-Trp were also formed. The bottom two spectra were collected on commercial standard samples.

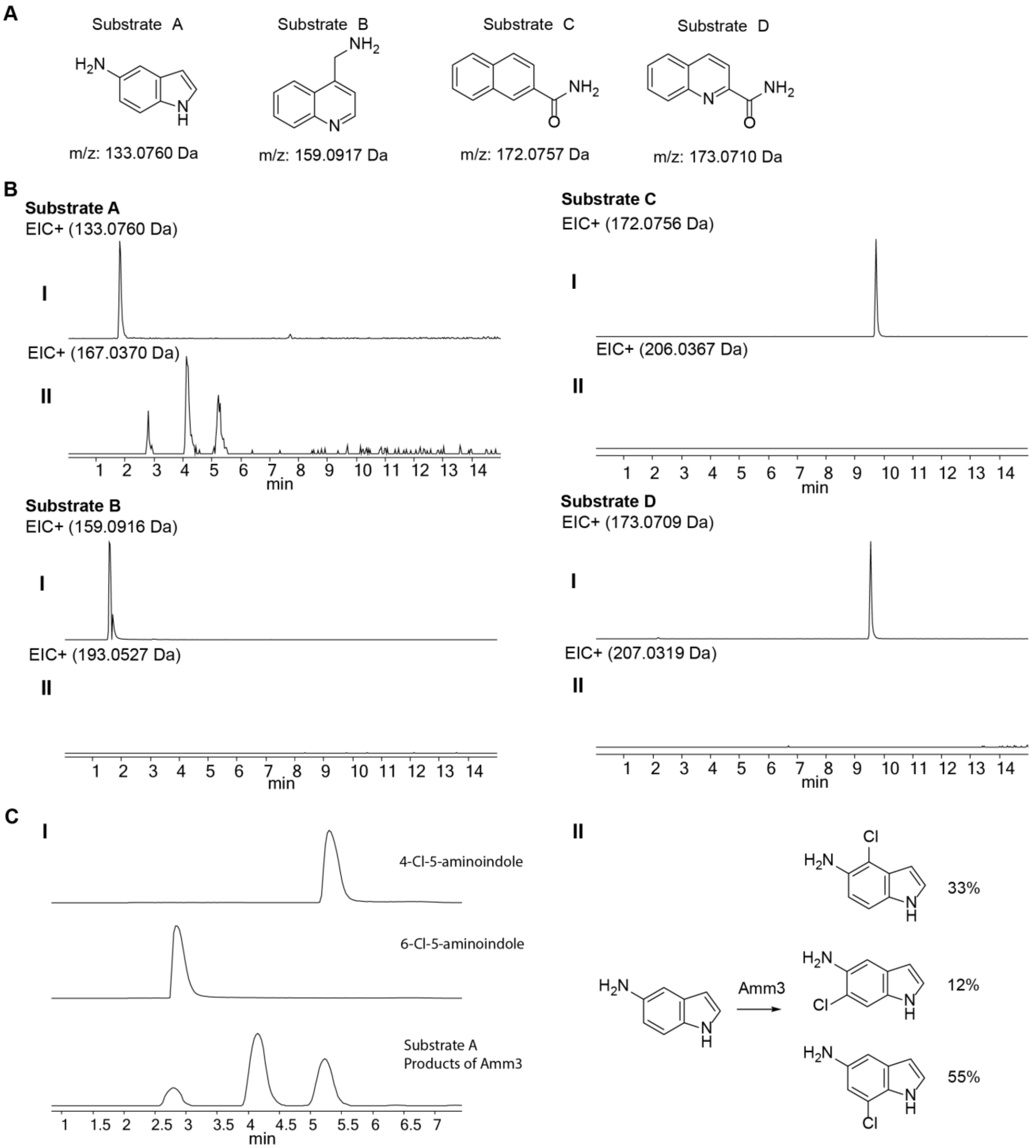

Figure S16. Substrate scope for chlorination catalyzed by Amm3. (A) Indole and quinoline analogs tested as potential substrates for Amm3. (B) Only the indole containing substrate A was processed by Amm3; bulkier quinoline or naphthalene containing compounds were not chlorinated by Amm3. (C) Further examination of the products of the reaction of substrate A with Amm3 by LC-MS (panel I) revealed that 4-Cl-5-aminoindole, 6-Cl-5-aminoindole, and putatively 7-Cl-5-aminoindole were produced (panel I and II).

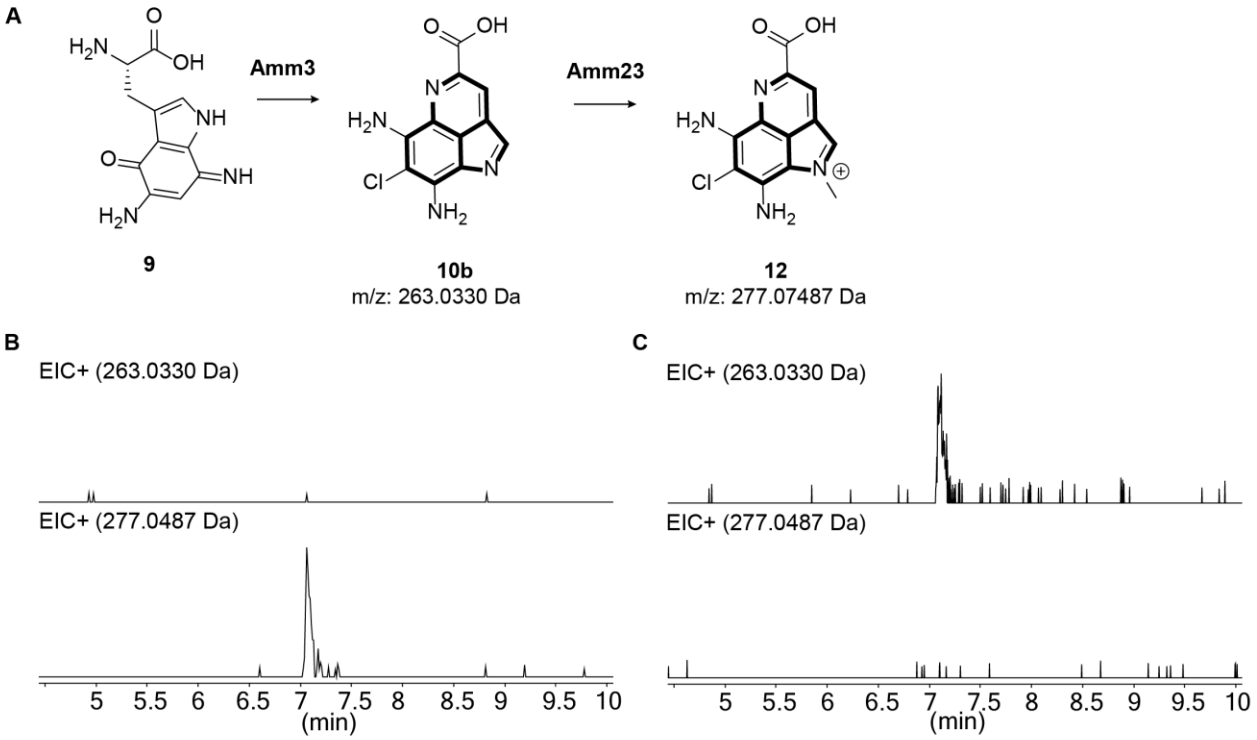

Figure S17. In vitro reaction of Amm23 with intermediate **10b**. (A) The product of the reaction of **9** with Amm3 produces **10b**. The reaction mixture was desalted and used in separate reactions with Amm23: (B) LC-MS analysis of a reaction containing **10b**, SAM, and Amm23. (C) LC-MS analysis of a reaction containing **10b** and SAM but without Amm23.

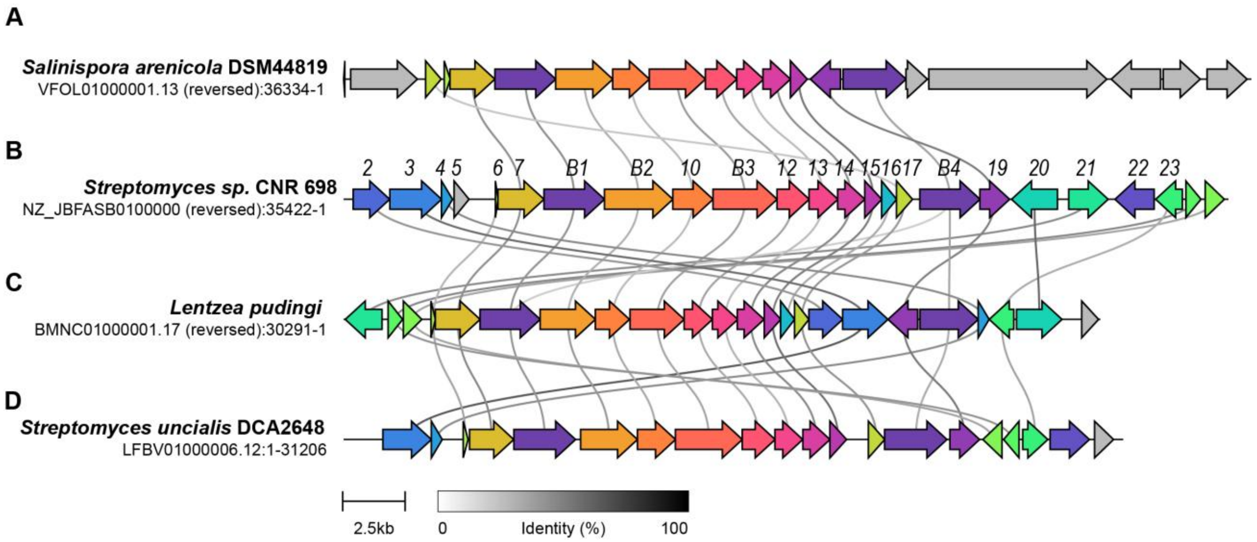

Figure S18. Comparison of the BGC from *Streptomyces sp.* CNR698 that produces ammosamide with the lymphostin producing BGC from *Salinospora arenicola* DSM44819,[6] with the putative ammosamide-like-producing orthologous BGC from *Lentzea pudingi* that lacks an *amm22* gene homolog, and with the ammosester-producing BGC from *S. uncialis* DCA2648.[9] The comparison was generated using Clinker.

References

(1) Ramos Figueroa, J.; Zhu, L.; van der Donk, W. A. Unexpected transformations during pyrroloiminoquinone biosynthesis. *J. Am. Chem Soc.* **2024**, *146*, 14235-45.
(2) Payne, J. T.; Andorfer, M. C.; Lewis, J. C. Regioselective arene halogenation using the FAD-dependent halogenase RebH. *Angew. Chem. Int. Ed.* **2013**, *52*, 5271–4.
(3) Ortega, M. A.; Cogan, D. P.; Mukherjee, S.; Garg, N.; Li, B.; Thibodeaux, G. N.; Maffioli, S. I.; Donadio, S.; Sosio, M.; Escano, J.; Smith, L.; Nair, S. K.; van der Donk, W. A. Two flavoenzymes catalyze the post-translational generation of 5-chlorotryptophan and 2-aminovinyl-cysteine during NAI-107 biosynthesis. *ACS Chem. Biol.* **2017**, *12*, 548-57.
(4) Vrtis, J. M.; White, A. K.; Metcalf, W. W.; van der Donk, W. A. Phosphite dehydrogenase: A versatile cofactor-regeneration enzyme. *Angew. Chem. Int. Ed.* **2002**, *41*, 3257.
(5) Jordan, P. A.; Moore, B. S. Biosynthetic pathway connects cryptic ribosomally synthesized posttranslationally modified peptide genes with pyrroloquinoline alkaloids. *Cell Chem. Biol.* **2016**, *23*, 1504-14.
(6) Miyanaga, A.; Janso, J. E.; McDonald, L.; He, M.; Liu, H.; Barbieri, L.; Eustaquio, A. S.; Fielding, E. N.; Carter, G. T.; Jensen, P. R.; Feng, X.; Leighton, M.; Koehn, F. E.; Moore, B. S. Discovery and assembly-line biosynthesis of the lymphostin pyrroloquinoline alkaloid family of mTOR inhibitors in *Salinispora* bacteria. *J. Am. Chem. Soc.* **2011**, *133*, 13311-3.
(7) Ting, C. P.; Funk, M. A.; Halaby, S. L.; Zhang, Z.; Gonen, T.; van der Donk, W. A. Use of a scaffold peptide in the biosynthesis of amino acid-derived natural products. *Science* **2019**, *365*, 280-4.

(8) Abramson, J.; Adler, J.; Dunger, J.; Evans, R.; Green, T.; Pritzel, A.; Ronneberger, O.; Willmore, L.; Ballard, A. J.; Bambrick, J.; Bodenstein, S. W.; Evans, D. A.; Hung, C.-C.; O'Neill, M.; Reiman, D.; Tunyasuvunakool, K.; Wu, Z.; Žemgulytė, A.; Arvaniti, E.; Beattie, C.; Bertolli, O.; Bridgland, A.; Cherepanov, A.; Congreve, M.; Cowen-Rivers, A. I.; Cowie, A.; Figurnov, M.; Fuchs, F. B.; Gladman, H.; Jain, R.; Khan, Y. A.; Low, C. M. R.; Perlin, K.; Potapenko, A.; Savy, P.; Singh, S.; Stecula, A.; Thillaisundaram, A.; Tong, C.; Yakneen, S.; Zhong, E. D.; Zielinski, M.; Žídek, A.; Bapst, V.; Kohli, P.; Jaderberg, M.; Hassabis, D.; Jumper, J. M. Accurate structure prediction of biomolecular interactions with AlphaFold 3. *Nature* **2024**, *630*, 493-500.
(9) Luo, J.; Yang, D.; Hindra; Adhikari, A.; Dong, L.-B.; Ye, F.; Yan, X.; Rader, C.; Shen, B. Discovery of ammosesters by mining the *Streptomyces uncialis* DCA2648 genome revealing new insight into ammosamide biosynthesis. *J. Ind. Microbiol. Biotechnol.* **2021**, *48*, kuab027
